# Proteomic analysis of sialoliths from calcified, lipid and mixed groups as a source of potential biomarkers of deposit formation in the salivary glands

**DOI:** 10.1186/s12014-023-09402-3

**Published:** 2023-03-22

**Authors:** Natalia Musiał, Aleksandra Bogucka, Dmitry Tretiakow, Andrzej Skorek, Jacek Ryl, Paulina Czaplewska

**Affiliations:** 1 Intercollegiate Faculty of Biotechnology UG&MUG, University of Gdańsk, Abrahama 58, 80-307 Gdańsk, Poland; 2 Institute of Biochemistry, Medical Faculty, Justus Liebig University of Giessen, Friedrichstrasse 24, 35392 Giessen, Germany; 3 Department of Otolaryngology, Faculty of Medicine, Medical University of Gdańsk, Smoluchowskiego 17, 80-214 Gdańsk, Poland; 4 Division of Electrochemistry and Surface Physical Chemistry, Faculty of Applied Physics and Mathematics, Gdańsk University of Technology, G. Narutowicza 11/12, 80-233 Gdańsk, Poland

**Keywords:** Sialolithiasis, Salivary stones, Mass spectrometry, SWATH-MS, Functional analysis

## Abstract

Salivary stones, also known as sialoliths, are formed in a pathological situation in the salivary glands. So far, neither the mechanism of their formation nor the factors predisposing to their formation are known despite several hypotheses. While they do not directly threaten human life, they significantly deteriorate the patient’s quality of life. Although this is not a typical research material, attempts are made to apply various analytical tools to characterise sialoliths and search for the biomarkers in their proteomes. In this work, we used mass spectrometry and SWATH-MS qualitative and quantitative analysis to investigate the composition and select proteins that may contribute to solid deposits in the salivary glands. Twenty sialoliths, previously characterized spectroscopically and divided into the following groups: calcified (CAL), lipid (LIP) and mixed (MIX), were used for the study. Proteins unique for each of the groups were found, including: for the CAL group among them, e.g. proteins from the S100 group (S100 A8/A12 and P), mucin 7 (MUC7), keratins (KRT1/2/4/5/13), elastase (ELANE) or stomatin (STOM); proteins for the LIP group—transthyretin (TTR), lactotransferrin (LTF), matrix Gla protein (MPG), submandibular gland androgen-regulated protein 3 (SMR3A); mixed stones had the fewest unique proteins. Bacterial proteins present in sialoliths have also been identified. The analysis of the results indicates the possible role of bacterial infections, disturbances in calcium metabolism and neutrophil extracellular traps (NETs) in the formation of sialoliths.

**Supplementary Information:**

The online version contains supplementary material available at 10.1186/s12014-023-09402-3.

## Introduction

Sialolithiasis is a rare, little-known, and poorly described disease. It affects 1 per 10 000–30 000 individuals per year [1, 2] and accounts for about 30% of salivary gland disorders [1]. This disease is a pathological condition connected with the formation of deposits called salivary stones or sialoliths in the salivary ducts or salivary glands. They are formed mainly in the submandibular (4–6:1) than the parotid and sublingual salivary glands [1, 3, 4]. About 25% of the patients with sialolithiasis have formed at least two salivary stones [5, 6].

**Fig. 1:**
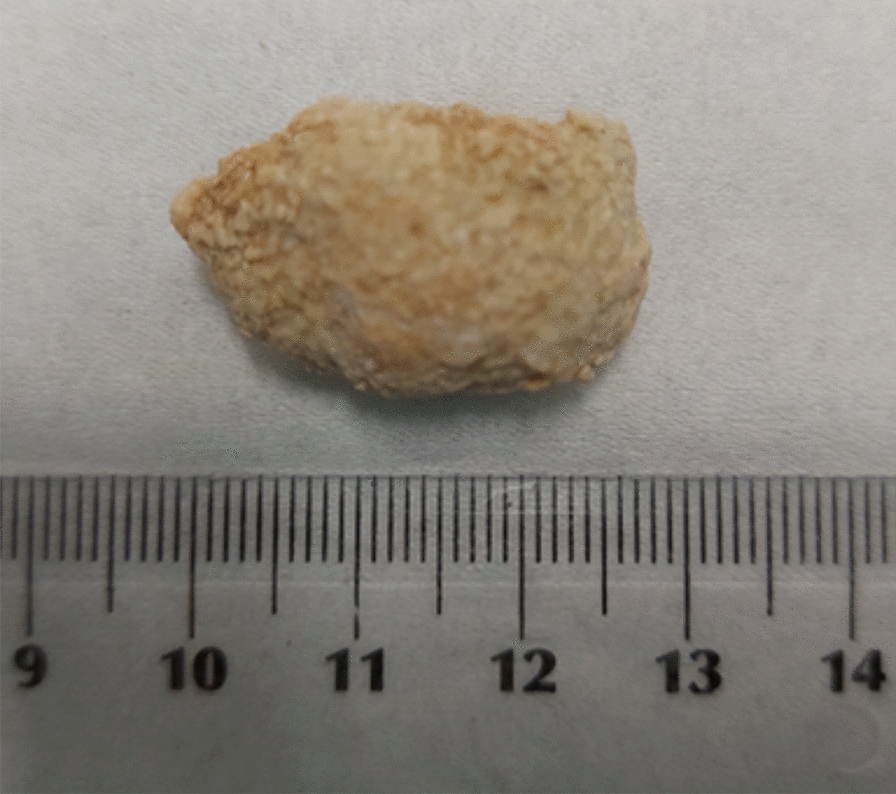
Salivary stone with diameter equal almost 30 mm

The problem for the patient arises when sialolith obstructs the salivary gland duct and interferes with saliva flow. During each meal, there is sudden pain and swelling in the salivary gland area, significantly worsening the patient's quality of life. The resulting inflammation of the salivary gland can become bacterial superinfection and purulent inflammation. Unfortunately, only surgical methods of treating sialolithiasis are known today [1–4, 7]. Unlike kidney stones or gallbladders, there are also no known methods of preventing sialolithiasis [8]. It is a severe problem because the sialolithiasis tends to recur, so that surgical treatment may be needed more than once. This kind of treatment can be risky, considering the possibility of injury to nearby structures, such as the marginal mandibular branch of the facial nerve and lingual nerve [9]. What is more, in some cases, the whole salivary gland must be removed. Besides, sialolithiasis can cause a feeling of discomfort because of its size. Most sialoliths’ diameter ranges between 2.1 and 10 mm, but there were noted cases when the diameter of one of the salivary stones was almost 30 mm (Fig. 1). According to the proposed by Tretiakow and coworkers classification [10] based on the spectroscopic studies of the stones, three types of sialoliths were proposed: calcified (CAL), organic/lipid (LIP), and mixed (MIX). Based on that classification, it can be supposed that CAL and LIP stones have different origins and development paths, while MIX is formed as CAL stones, and the other pathway of their growth passes as LIP stones. During these stages, controlling the balance of calcium and lipids is disturbed.

The mechanism of sialolith formation remains still unknown. At the same time, the number of hypotheses about their formation increased. According to one of the theories about sialolith formation, bacteria may influence the biocalcification process leading to the stone formation. Among the potential causes of the origin of sialolithiasis belong injury and inflammation within the salivary glands, precipitation of salts, dental and endocrine disease, microliths around which calcium crystals can deposit, the alkalinity of saliva causing precipitation of calcium phosphate, oral bacteria and food debris and its migrations [2, 11].

It suggests that the process is rather multifactorial than caused by one particular agent or event. We still do not have any in vitro or in vivo models to produce calculi and study their formation mechanism. There are predisposing factors which can influence sialolith formation: long winding course of the salivary gland duct (Wharton's duct), higher mucus concentration, reduced fluid intake, tobacco smoking, and some medications, which reduce the amount of secreted saliva [12–14]. As we can see, the salivary gland's secretory activity may significantly impact the biocalcification process in sialolithiasis. The secretion is controlled by the autonomous nervous system [15, 16]. When the saliva flow is reduced, the presence of leucocytes in saliva can be observed, as in patients with recurrent parotitis [17, 18]. Besides, the number of leucocytes can be increased because of bacteria presence [19–22]. Ongoing inflammation and salivary stasis can cause glandular dysfunction, atrophy, or sclerosis [23–25]. One research showed that neutrophil extracellular trap (NET) formation is essential for sialolith development [26]. We talk about the formation of NETs when the neutrophils externalise their chromatin connected with granular proteins, such as leucocytes in saliva [27]. The formation of NETs is an inflammatory response to the contact of neutrophils with crystals such as calcium salts [28], cholesterol [29] and urate [30], also by pH variations [31], foreign bodies [32] and the presence of bacteria [33]. Because NETs tend to aggregate, there is the formation of aggNETs [34–38], which play the role of “glue”, sticking proteins and calcium crystals, leading to the formation of macroscopic sialoliths. Neutrophil extracellular traps were noted as an inducer of the formation of depositions in other organs in vivo [29, 35]. Targeting NETs formation may become a valuable instrument for preventing the development of salivary stones [26]. This research team used DNA staining and immunostaining to detect potential biomarkers, but MS-based methods are more accurate and universal. Recently, Kraaij and co-workers, in a pilot study on sialoliths, identified, using electrophoretic and mass spectrometric methods, proteins that may be involved in the process of deposit formation in the salivary glands [39]. These included proteins involved in defence against infection, such as IgA, MUC7, and lysozyme. They noticed the presence of lactoferrin secreted by neutrophils, which is associated with NET.

There are few studies analysing the organic components of salivary glands stone in the literature, and further studies of the ultrastructure and protein composition of sialoliths are necessary to understand the mechanisms of their formation and development. Early studies showed that the epithelial cells of the salivary glands, where mainly sialoliths are formed, can secrete multifunctional proteins and peptides as the main components of saliva. These components are responsible for buffering, digestion, mineralisation, lubrication, and tissue coating and possess antimicrobial properties (secrete antimicrobial proteins and peptides, antibodies, and cytokines) [13]. Among the main groups of active compounds, histatins, acidic and glycosylated PRPs (proline-rich proteins), and phospho-forms of statherin are present [40, 41]. The level or structure of proteins secreted by salivary glands or bacterial infection can affect the process of controlling the balance of calcium and lipids, leading to the formation of sialoliths.

Fusconi et al. showed the biofilm-type bacterial aggregates accumulated in cores of salivary stones, which were surrounded by organic outer layers containing glycoproteins. Furthermore, biofilm was detected as a pathogenic factor in the formation of stones in the kidney and gallbladder [42–45]. Identified bacteria species are part of the oral microbiome. Summarizing several pieces of research, the most commonly detected bacteria in salivary stones are *Staphylococcus, Streptococcus, Peptostreptococcus sp., Actinomyces viscosus, Bacillus cereus, Eikenella corrodens, Fusobacterium nucleatum, Gemella sanguinis, Haemophilus parainfluenzae, Neisseria subfava, Propionibacterium acnes, Pseudomonas aeruginosa* and *Serratia marcescens* [22, 42, 46, 47].

Our first published research [48], based on a relatively small sialoliths group (five stones), showed some proteins with a critical role during the formation of sialoliths. One of them was a group of cystatins (CYST1-5). These proteins are responsible for many processes: regulating the activity of enzymes from the cathepsin group, protein degradation [49], response to the presence of pathogenic bacteria, control of calcium-phosphate homeostasis, causing in this way the formation of the pellicle and remineralisation processes of the teeth [50]. Some cystatins are also able to oligomerise and can bind other proteins causing coprecipitation in the salivary stones, analogous to the cerebral deposits in cerebral haemorrhages [23, 51–53] peptide, lysozyme C, mucin-7, immunoglobulins, S100-A9 and A100-A8. Statherin was identified during proteomic analysis, both qualitatively and quantitatively. This protein stabilises saliva supersaturated with calcium salts by inhibiting the precipitation of calcium phosphate salts [54]. A similar situation is in the case of one of the previous research projects that showed that mucin 8 (MUC8) could be an exciting candidate biomarker for salivary stone disease [55]. It was noted that MUC8 is upregulated during inflammatory processes in the respiratory tract and the pathogenesis of salivary stone diseases [56]. Only one potential biomarker was tested during this study, while MS-based proteomics performing only one experiment can detect many potential biomarkers.

In order to deepen our knowledge of the protein and bacterial composition of salivary gland stones, we performed a proteomic analysis on a group of 20 stones from patients diagnosed with sialolithiasis. We used the FASP digestion procedure proposed in the previous work for this biological material, obtaining more human and bacterial protein identifications. The SWATH-MS quantitative analysis of the proteins from sialoliths connected with functional analysis was performed for all samples with comprehensive data analysis to identify potential biomarkers of sialolithiasis.

## Methodology

### Collecting samples

All 20 salivary stone samples were collected from patients treated in the Department of Otolaryngology at the Medical University of Gdańsk. All patients were included in the study only after signing the necessary written consent and approval by the Independent Bioethics Commission at the Medical University of Gdańsk. First, they were analysed using spectroscopic methods and then divided into 3 three groups according to the proposed classification [10]. There are 6 calcified stones (CAL), 4 lipid stones (LIP) and 10 mixed stones (MIX) (Table 1). Such designations will be used in the remainder of the manuscript. Because they were tested earlier, all of them were in pieces; there was no sialolith in one piece. Collecting the samples was optimised and standardised according to the applicable routine protocol from the Clinic of Otolaryngology with the Department of Oral and Maxillofacial Surgery at The University Clinical Centre in Gdańsk. Sialoliths were removed during endoscopic, transoral or transcervical surgery. After that, the salivary stone samples were washed with the use buffer (25 mM NH_4_HCO_3_) and then transported in sterile falcon tubes to the Intercollegiate Faculty of Biotechnology of the University of Gdańsk and Medical University of Gdańsk and stored at 80 °C for further experiments.

**Table 1:** Division of clinical samples into 3 separate groups based on spectroscopic studies: calcified (CAL) group, lipid (LIP) group and mixed (MIX) group

	CAL group	LIP group	MIX group
Sample No.	1,4,5,9 (outer layers),10,12	3,11,19,20	2,6,13,14,15,16,17,18,21, 22

### Construction of the spectral library for CAL, LIP and MIX sialoliths

The used approaches treating salivary stone samples were based on protocols described in the publication with the results of trial proteomic analysis of sialoliths [48]. The first step included crushing the stones into the powder in a mortar. The portion of the 50 mg of powdered sialolith was treated with 250 µl lysis buffer (1% SDS, 100 mM Tris–HCl pH 8,0, 50 mM DTT), incubated at 95 °C for 20 min with mixing and left in the fridge at 4 °C. The incubation with occasional mixing lasted one day, and then the supernatant was collected. The procedure was repeated three times, finally obtaining a supernatant fraction (three lysis buffer fractions combined). For the supernatant fraction, standard FASP digestion was performed on a 10 kDa membrane [57]. In all cases, proteins were digested overnight at 37 °C after adding trypsin. Obtained peptide fractions were prepared for MS analysis by final clean-up on C18 (exchange disks 3 M EmporeTM) StageTips according to the protocol described by Rappsilber [58].

To build a well-developed spectral library and maximise the number of quantified proteins, pooled protein extract from sialolith samples was prepared and intended for protein separation in gel and after digestion according to the standard FASP approach for basic pH chromatographic separation. The final spectral library intended for SWATH-MS analysis consisted of.

(i) Spectra recorded in the information-dependent format (IDA) for protein fractions after separation in gel (SageELF) digested according to FASP;
(ii) Peptide fractions after digestion with FASP and chromatographic separation in alkaline pH;
(iii) Individual IDA spectra recorded for each sialolith sample studied in this work.

#### Chromatographic separation

Basic pH HPLC fractionation of tryptic peptides was performed based on the protocol described by Lewandowska [59]. Separation was carried out using Nexera XR HPLC System with the PDA detector (Shimadzu, Kyoto, Japan) and Jupiter Proteo 90 Å column (4 μm, 250 × 2.6 mm, Phenomenex, Torrance, CA). Used buffers—A: 100 mM ammonium bicarbonate pH 8, and B: 100% ACN. The flow rate was 1 ml/min, and the gradient was 0–50% B. During a 2 h chromatographic run, 60 fractions of 2 ml each were manually collected. Chromatographic separation was repeated three times. All fractions from separate runs were combined at the same time points, and then they were evaporated to dryness in SpeedVac and dissolved in 100 µl of 60% ACN with the addition of 1% AA. Before LC–MS/MS measurements, samples were concentrated into 30 µl fractions ready for MS analysis.

#### In gel separation

The second approach was automated electrophoretic separation using SageELF automatic system (Sage Science), which allows protein separation into 12 fractions and direct elution from the gel. The protein sample was applied to the commercial, ready-to-use 3% SDS-Agarose Gel Cassette (ELP3010, Sage Science), and the separation process was automated. This cassette separates proteins in the 10–300 kDa range. All the necessary solutions (Loading buffer) and the marker are supplied with the cassettes in the set. The sample was dissolved in a loading solution to obtain a protein concentration of 200 μg protein in 26 μl, and 10 μl loading solution with fluorescent Marker-03 was added to the sample, and the sample was mixed. The reduction step with the addition of DTT by heating to 85 °C was omitted as the sample was previously treated with lysis buffer and heated. The proteins prepared this way were introduced into a 3% SDS-Agarose cassette. Separation (size-based mode) proceeded through 1 h 20 min and then through 30 min automatic elution of proteins from the gel. The system was controlled by SageElf software version 1.08. In the end, the fractions were collected from the cassette wells. This step separated the portion of 350 μg of protein pooled sample. Then, all obtained fractions were digested separately according to FASP and cleaned up (StageTips) as described above.

#### LC–MS/MS analysis

LC–MS/MS analysis was performed on Triple-TOF 5600 + mass spectrometer (AB Sciex LLC, Framingham, MA, USA) connected with Ekspert MicroLC 200 Plus System (Eksigent, Dublin, CA, USA). The Analyst TF 1.7.1 software (SCIEX) controlled the whole system. The chromatographic gradient for each MS run was 11–42% B (A: H_2_O + 0,1% FA; B: 100% can + 0,1% FA) in 60 min. ChromXP C18CL column (3 µm, 120 Å, 150 × 0.3 mm) was used to perform the chromatographic separation. The spectra were registered in information-dependent acquisition (IDA) mode to perform qualitative analysis and build the library. Each cycle comprised precursor spectra accumulation in 100 ms in the range of 400–1200 m/z followed by top 20 precursor ion spectra accumulation in 50 ms in the range of 100–1800 m/z, resulting in a total cycle time of 1.15 s. Formerly fragmented precursor ions were dynamically excluded.

### Qualitative analysis

The results of MS analysis performed in IDA mode were analysed in PeaksSTUDIO software with the following settings: instrument—TripleTOF, fragmentation method—CID, acquisition—IDA, precursor mass tolerance error—0.1 Da using monoisotopic mass, fragment ion tolerance—0.2, digestion—trypsin, reduction and alkylation of proteins (fixed modification—carbamidomethylation; variable modification—acetylation N-term, oxidation M), maximum allowed variable PTM per peptide—3. The raw data generated during MS analysis had to be converted from.wiff format to.mzML format with MSconvert software [60]. First, the data were analysed against the *Homo sapiens* database (Uniprot, 15.11.2021), and then, using the Multi-Round Search function, were analysed against the entire bacterial database (Uniprot, 15.11.2021). It allowed for identifying proteins from different organisms in one experiment.

### Digestion of clinical samples of CAL, LIP and MIX sialoliths for SWATH analysis

The portion of the 50 mg of powdered sialolith was treated with 250 µl lysis buffer (1% SDS, 100 mM Tris–HCl pH 8,0, 50 mM DTT), incubated at 95 °C for 20 min with mixing and left in the fridge at 4 °C with occasional mixing for 3 days. After this time, the supernatant was not collected – the whole fraction with powder and supernatant was used in the next step for supernatant/pellet digestion. For the supernatant/stone powder mix, FASP digestion was performed by introducing a pellet of stones and the supernatant onto a membrane; the stone pellets did not interfere with filtration and did not clog the membrane. Before LC–MS analysis, the samples were subjected to final clean-up (StageTips) as described above.

### Quantitative analysis

To carry out quantitative analysis, the SWATH experiments were performed. The equalized frequency of precursor ions and coverage of the precursor mass range of 400–1200 m/z was used to construct the set of 25 transmission windows of variable width with SwathTUNER software [61]. The collision energy for each window was calculated for + 2 to + 5 charged ions centred upon the window with a spread of five. The SWATH-MS survey scan was acquired in the range covered by constructed windows at the beginning of each cycle with an accumulation time of 50 ms. Following SWATH-MS/MS spectra, product ion scans were collected in the range of 100 to 1800 m/z in 40,014 ms, which resulted in a total cycle time of 10999 s. Spectra were registered in 3 technical replications in data-independent acquisition (DIA) mode for each sample [62].

Data were analysed in PeakView 2.2 software (SCIEX), and DIA spectra were processed against the created sialoliths spectral library, which was constructed using ProteinPilot 4.5 software (Sciex; *Homo sapiens* database, Uniprot, 01.02.2022) from DDA spectra for all clinical samples, pooled ones (HPLC and SageELF fractionated). After processing all of the clinical samples registered in DIA mode in PeakView software according to settings described by Lewandowska [62], SWATH data were generated.

The mass spectrometry proteomics data have been deposited to the ProteomeXchange Consortium (http://proteomecentral.proteomexchange.org) via the PRIDE partner repository [63] with the dataset identifier PXD039381.

### Statistical and enrichment data analysis

First, SWATH results were analysed to identify statistically significant proteins. First, final files from PeakView software were exported to MarkerView 1.2.1.1 software (Sciex). Data were normalised using the total area sums (TAS) approach. Then, the output table was exported to Perseus 1.6.13 software (MaxQuant) [64] for the normalisation of data (log2(x)), and the statistical tests were performed to get the q-value (adjusted p-value; q-value < 0.05) for each protein. This software also calculated fold change values (FC) for each protein and sample, so sets of proteins were obtained, which are up-regulated and down-regulated. Control was pooled sample. Based on the results of the quantitative analysis, the enrichment analysis was carried out. For this purpose, several bioinformatic tools were used: STRING 11.5 [65], Gene Set Annotation (GSAn) [66] and g:Profiler [67]. The results were visualised in the Cytoscape 3.9.1 software [68]. Venn diagram was generated with the use InteractiVenn tool [69].

## Results

**Fig. 2:**
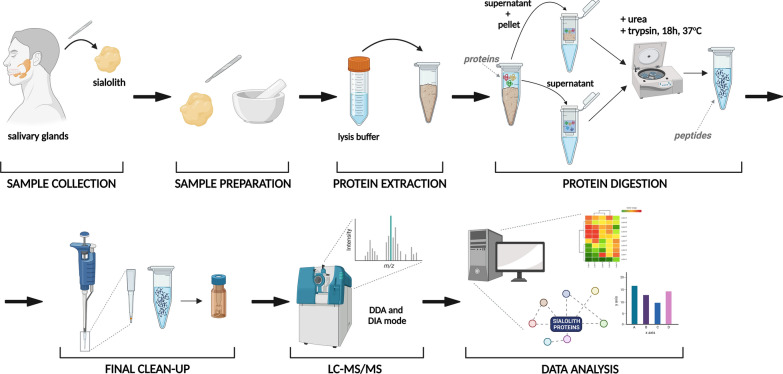
The workflow of the study. Collected sialolith samples were divided into separate layers and crushed. Extracted proteins were digested using the FASP approach. Desalted peptides were ready for LC–MS/MS analysis in DDA and DIA modes. Results were statistically and functionally analysed [70]

The studies were performed on a set of clinical samples including 20 sialoliths, which were divided into 3 groups based on spectroscopic studies: 6 calcified stones, 4 lipid stones and 10 mixed stones. The diagram of the entire process, starting from a stone sample and ending with data analysis, is shown in Fig. 2. For the qualitative analysis and, simultaneously, for the creation of the spectral library required for the quantitative SWATH analysis, the pooled clinical samples were processed (see materials and methods), and the IDA spectra were recorded. In addition, a three-stage enrichment of the library was carried out—the first step by using alkaline pH chromatographic separation of trypsin-digested peptides in FASP methodology. The second type of enrichment involved the separation of proteins extracted from stones on a gel into 12 fractions in the SageELF apparatus, which performs automatic elution of each fraction. Spectra in the IDA format were recorded for each fraction from the chromatographic and gel separation and individual clinical samples. Trypsin-digested peptides were separated using HPLC chromatography at alkaline pH, which increased from about 200 IDs to 694 and made the most outstanding contribution to the spectral library. In gel separation combined with automatic elution in the SageELF system (see materials and methods) combined with further digestion (FASP) of the thirteen fractions obtained increased the ID number in a library to 794 human proteins.

For each clinical sample, the possibility of digesting the powdered material directly on the membrane was checked according to the FASP methodology. The aim was to obtain as much protein extract as possible for digestion and the possibility of direct digestion on the sialolith pellet. Due to its structure, the powder did not block the membrane, and it was possible to perform all standard FASP steps for each sample freely. The two protocols were compared quantitatively, and the results were similar. Fragmentation spectra were analyzed using PeaksStudio (qualitative analysis) and Protein Pilot (spectral library for SWATH-MS analysis).

### Qualitative analysis

First, the data were analysed against the *Homo sapiens* database and then, using the Multi-round search function in PeaksStudio against the entire bacterial database. This step allowed the identification of 313 proteins at 1% FDR with a minimum of 2 different peptides. Comparing the number of identified proteins for both groups was obtained 162 human proteins and 151 bacterial proteins. Proteins were also grouped, and 134 groups of proteins were detected—93 groups contained human proteins, and 41 groups contained bacterial proteins (Additional file 1: Figure S1).

#### Bacterial proteins

**Fig. 3:**
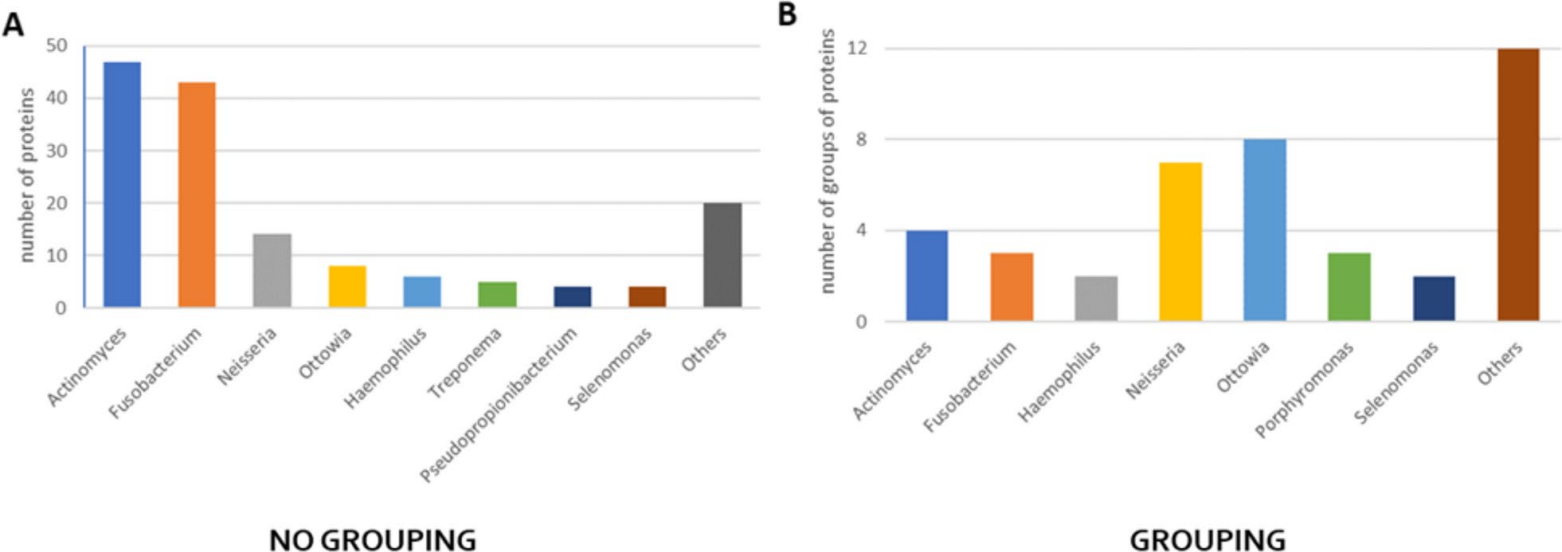
Chart presenting the number of proteins (**A**) and several groups of proteins (**B**) at 1% FDR with the minimum of 2 different peptides detected from a different genus of bacteria in all clinical samples

Regarding bacterial proteins, the highest number was identified in samples 3 and 11, which belong to LIP group, 12 from CAL group and 22 from MIX group. They were not identified only in 3 samples: 1 lipid stone (sample 19) and 2 mixed stones (samples 16 and 18). In Fig. 3 there are seen the genus of bacteria and the number of proteins identified from them with PeaksSTUDIO software, both when the proteins were not in the group and grouped. There are the common for all salivary stone types of bacteria groups, from which the most proteins were identified: *Actinomyces, Fusobacterium, Neisseria* and *Ottowia*.

Analysing the species of bacteria from which the proteins were detected, 44 species were identified in total in all of the samples (Additional file 1: Table S1).

Using the Venn diagram, groups of unique species for each type of sialolith sample and a group of common species for all 3 types were discerned (Table 2). There are 6 bacteria species unique for calcified salivary stones, 7 for lipid and mixed sialoliths. 13 are common for all 3 types of salivary stones.

**Table 2:** Unique species of bacteria, for which proteins were identified in different types of sialolith samples

Unique species for CAL sialoliths	Unique species for LIP sialoliths	Unique species for MIX sialoliths	Common species for CAL, LIP and MIX sialoliths
*Capnocytophaga sp. oral*	*Desulfobulbus oralis*	*Aggregatibacter aphrophilus*	*Actinomyces glycerinitolerans*
*Peptostreptococcus stomatis*	*Fretibacterium sp.*	*Haemophilus haemolyticus*	*Actinomyces johnsonii*
*Porphyromonas sp. oral*	*Fusobacterium canifelinum*	*Neisseria macacae*	*Actinomyces naeslundii*
*Selenomonas noxia*	*Fusobacterium hwasookii*	*Neisseria sicca*	*Actinomyces oris*
*Selenomonas sp. oral*	*Fusobacterium periodonticum*	*Neisseria sp. oral*	*Actinomyces sp. Oral*
*Treponema denticola*	*Pseudopropionibacterium propionicum*	*Rothia dentocariosa*	*Actinomyces viscosus*
	*Tannerella forsythia*	*Streptococcus mitis*	*Fusobacterium nucleatum*
			*Fusobacterium nucleatum subsp. Animalis*
			*Fusobacterium nucleatum subsp. Polymorphum*
			*Fusobacterium pseudoperiodonticum*
			*Fusobacterium sp.*
			*Neisseria bacilliformis*
			*Ottowia sp. Oral*

#### Human proteins

**Fig. 4:**
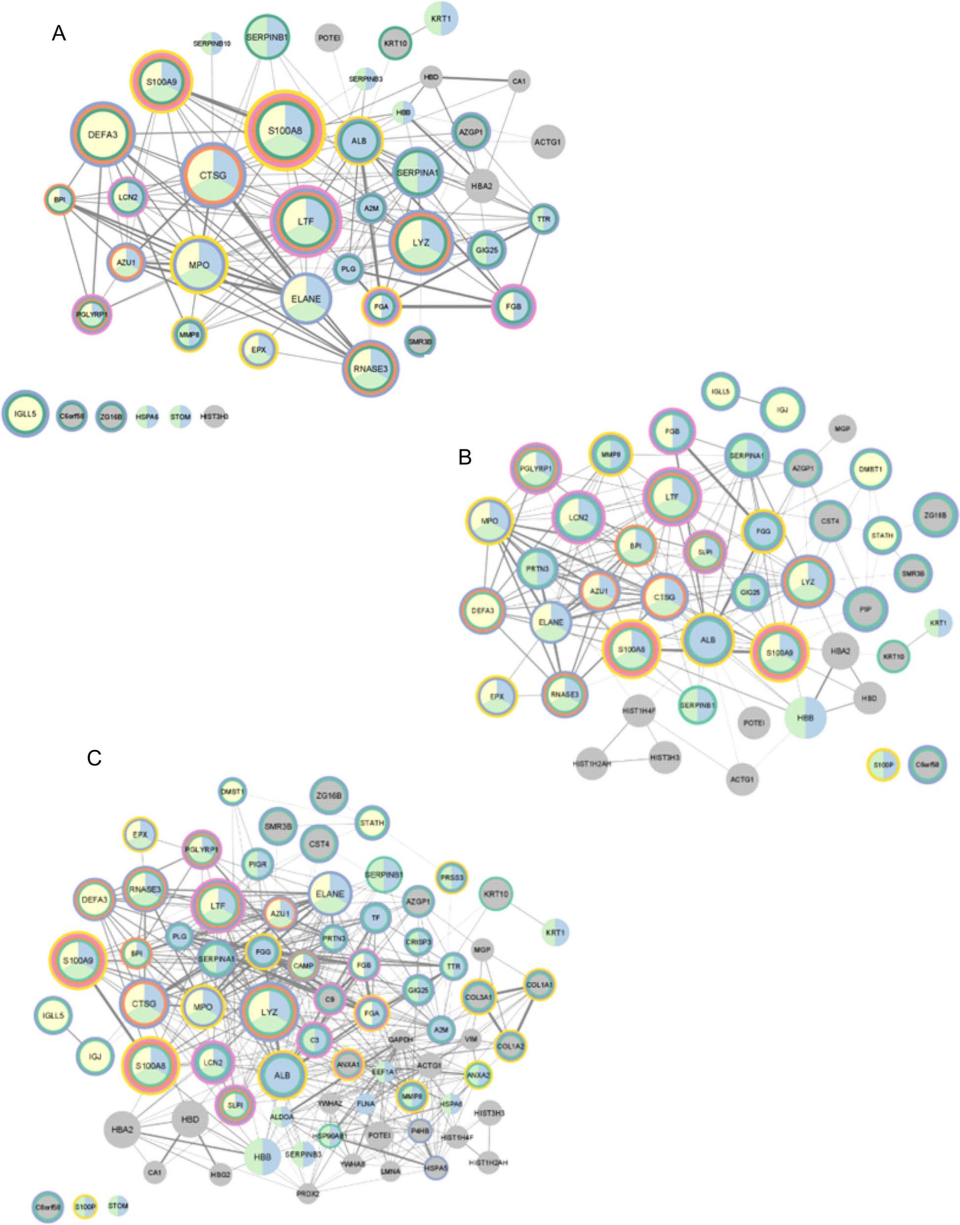
The Cytoscape visualisation of the STRING-generated network is composed of experimentally verified protein–protein interactions among the most common proteins identified in the CAL (**A**), LIP (**B)** and MIX (**C)** groups. The size of nodes corresponds with the frequency of occurrence of protein among all of the samples in the respectively groups; bigger nodes represent higher frequency, and smaller – ones represent lower frequency. The circles' colours correspond to Uniprot Annotated Keywords enrichment: green—secreted, orange—antimicrobial, blue—signal, pink—immunity, yellow—calcium. Fill colours correspond with Biological Process (GO) enrichment: yellow – defence response to the bacterium, blue – regulated exocytosis, green – neutrophil degranulation

To find the most common proteins in one group, human proteins identified in all of the salivary stone samples were compared among each group: calcified (CAL), lipid (LIP), and mixed (MIX). Only sets of proteins which were detected in each group were used. As a result, the received sets were composed of 42, 50 and 78 proteins, respectively. Proteins with the highest frequency between groups are neutrophil defensin 3 (DEFA3), protein S100-A9 (S100A9), protein S100-A8 (S100A8), cathepsin G (CTSG), myeloperoxidase (MPO), lactotransferrin (LTF), eosinophil cationic protein (RNASE3), and lysozyme C (LYZ). The Cytoscape visualisations of the STRING-generated network and enrichment analysis for the most common proteins identified in the CAL, LIP and MIX groups are presented in Fig. 4.

### Quantitative analysis

The results of MS analysis of clinical samples performed in DIA mode were analysed in PeakView 2.2 software using the constructed spectral library. The quantitative analysis allowed us to identify up-regulated and down-regulated proteins. For each analysed clinical sample, sets of proteins which were statistically significant (q < 0.05) and the log2(FC) ≥ 0.45 for up-regulated proteins and log2(FC) ≤ − 0.45 for down-regulated proteins were chosen. Relative quantitative analysis was performed considering the original classification of sialolith samples. To enrich the analysis and obtain additional information on unique and classified as significant based on the SWATH analysis, enrichment analysis was performed with the use of g:Profiler bioinformatic tool in the following annotation category: Gene Ontology (Biological Process, Cellular Component, Molecular Function), KEGG, Reactome and type of regulation: up-regulation, down-regulation or varied level of regulation of proteins among the samples in the group. Analyses were performed for each group and are available in the Supplementary Materials: CAL (Additional file 1: Figures S2), LIP (Additional file 1: Figures S3), and MIX (Additional file 1: Figures S4). To check the applicability of pooled samples as a control, principal component analysis (PCA) was conducted (Additional file 1: Figure S5).

**Fig. 5:**
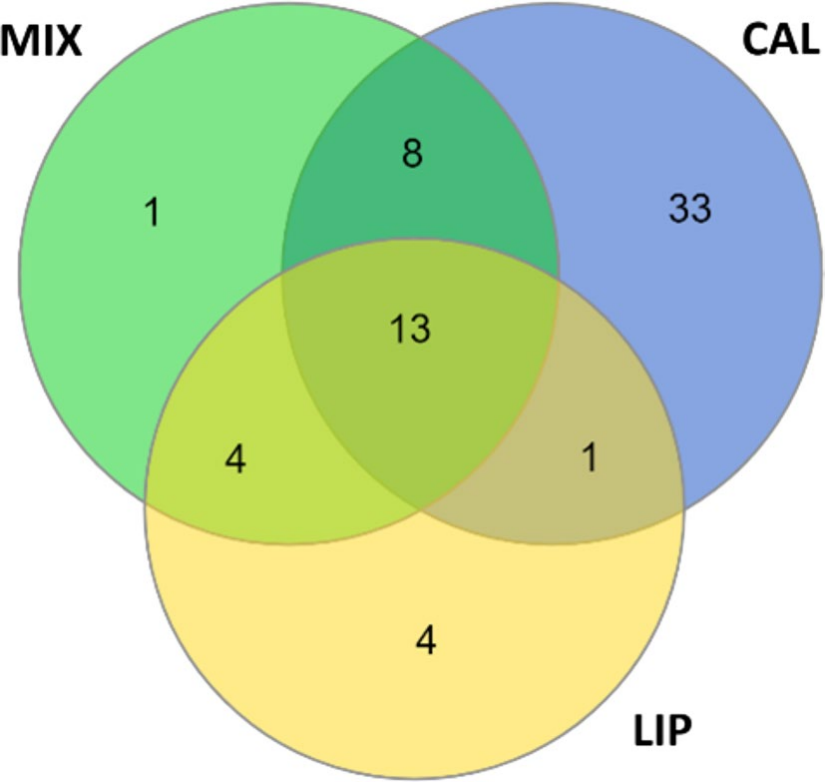
Venn diagram presenting the number of statistically significant proteins identified for more than 50% of samples from CAL, LIP and MIX groups

**Fig. 6:**
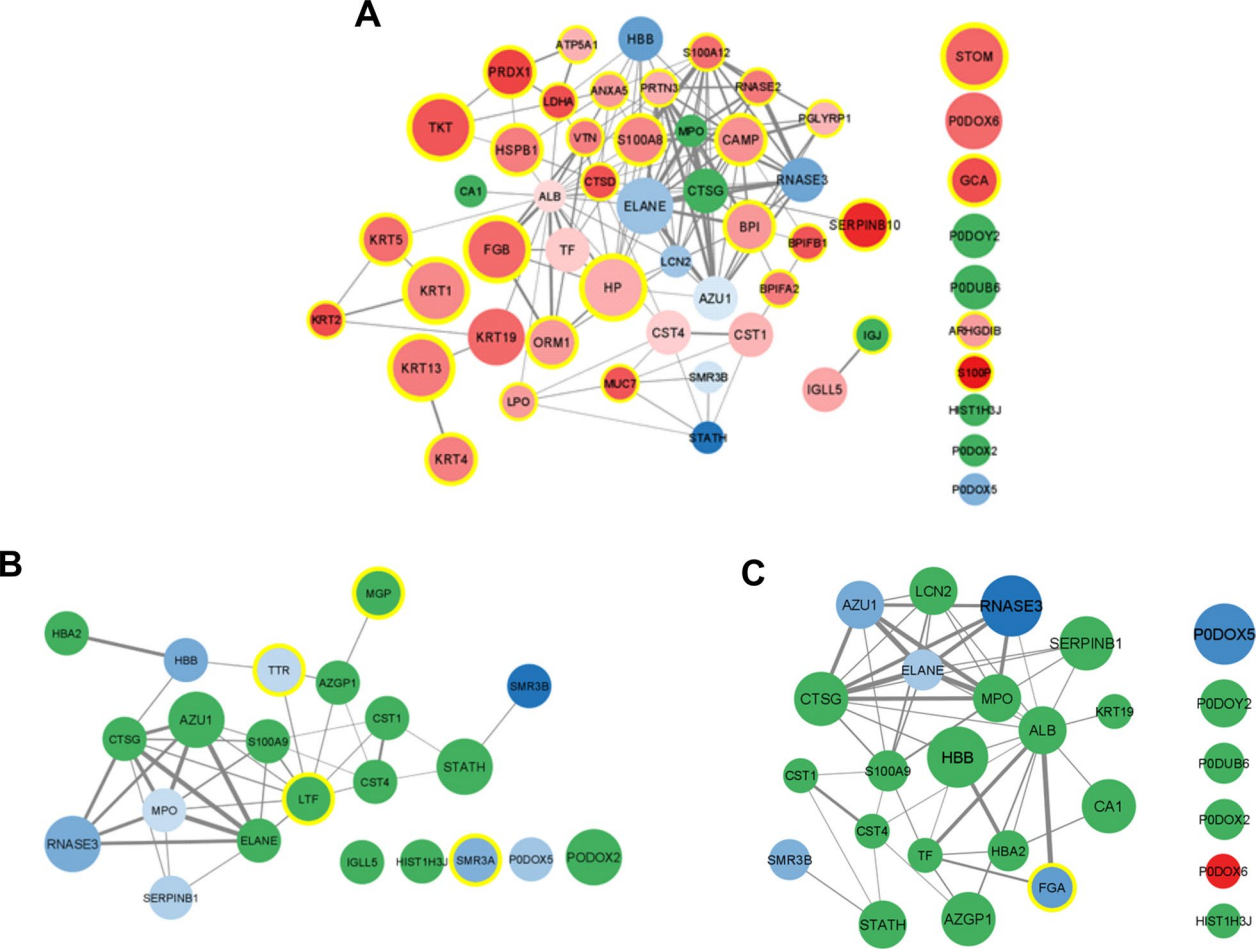
The Cytoscape visualisation of the STRING-generated network comprises experimentally verified protein–protein interactions among the most common proteins identified in (**A**) the CAL, (**B**) LIP and (**C**) the MIX group. The size of nodes corresponds with the frequency of occurrence of protein among all of the samples in the group; more significant nodes represent higher frequency, and smaller—ones represent lower frequency. The gradation of the fill corresponds with the median value of log2FC, presenting the type of regulation of protein: red—up-regulation of protein among the whole group and blue—down-regulation of protein. The darker the colour means, the more significant the difference. The green colour presents the case when the protein regulation level varies among the group's proteins. The yellow colour in the circles corresponds to the uniqueness of protein for the group

The comparison of the selected sets of the most common statistically significant proteins quantified for each group (it was assumed that these proteins must be present in more than 50% of samples in each group) is presented in Fig. 5 as a Venn diagram. Thanks to that, it was possible to detect the number of unique proteins for different groups. Based on the quantitative analysis, protein–protein interactions (STRING-generated network) were analysed for each studied group. The visualisation of the results, including quantitative data, was generated in the Cystoscape program (Fig. 6).

#### Calcified sialoliths

**Fig. 7:**
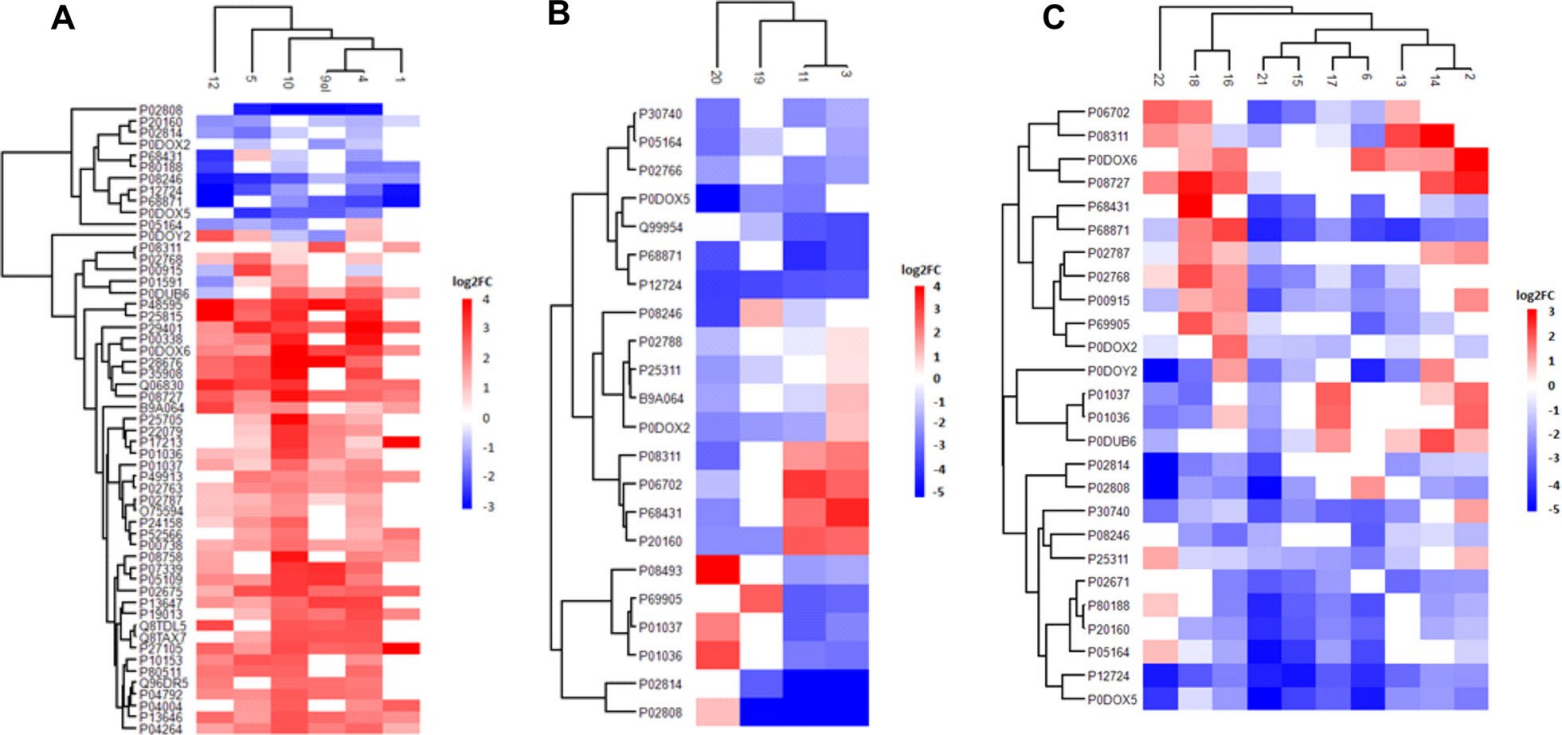
Heatmaps present the level of regulation of proteins, which are statistically significant (q < 0,05) and the log2FC ≥ 0,45 for up-regulated proteins and log2FC ≤ − 0,45 for down-regulated proteins for more than 50% of samples from CAL (**A**), LIP (**B**) and MIX (**C**) groups. The red colour corresponds with values of log(FC) for up-regulated proteins, and the blue corresponds with values of log(FC) for down-regulated proteins

In the case of calcified stones (CAL), 109 proteins were quantitatively analysed (Additional file 1: Table S2), among which up- and down-regulated proteins detected in more than 50% of samples from this group are presented in the form of a heatmap (Fig. 7A). A STRING-generated network presenting experimentally verified protein–protein interactions was prepared in Fig. 6A. The network presents the type of regulation of proteins, their frequency among the samples in the group and their uniqueness by marking the proteins with yellow circles for 33 quantified proteins, all unique for the CAL group. Almost all of them were up-regulated in each of the samples from the group. Only one protein—immunoglobulin J chain (IGJ) – was down-regulated in one sample (sample 12). The biggest group of unique up-regulated proteins is the keratin family (KRT1, KRT2, KRT4, KRT5, KRT13). The median values of fold change for these proteins are as follows: S100A8 = 4.30, S100A12 = 5.25, S100P = 11.76, S100A9 = 1.76 (protein S100A9 is not shown on the STRING-generated network, because it was not quantified in more than 50% of samples in CAL group—S100A9 was quantified in 3 calcified samples). Two common in salivary glands and saliva proteins from BPI fold-containing family (BPIFA2, BPIFB1), responsible for an antimicrobial activity causing changes in the cellular response to lipopolysaccharide by binding to LPS, are also up-regulated, pointing to the presence of bacteria [71, 72]. MUC7, typical for oral cavity protein, was up-regulated, similar to the case of MUC8 during inflammatory processes [55, 56]. Another unique CAL group protein was stomatin (STOM) which regulates ion channel activity in membranes, so a higher level of STOM can influence the concentration of calcium ions [73]. Changes in calcium concentration can also be associated with the up-regulation of transketolase (TKT). This enzymatic protein can form complexes with calcium [74]. The last 2 up-regulated unique proteins—haptoglobin (HP) and fibrinogen (FGB)—both have antimicrobial activity, which is connected with the presence of different bacteria [75, 76]. Non-unique but persistent protein is also up-regulated keratin 19 (KRT19), belonging to the mentioned earlier keratin family. Another protein common for the calcified group is down-regulated neutrophil elastase (ELANE), a serine proteinase secreted by neutrophils during inflammation. This protein has a high affinity to DNA. It can be found in neutrophil extracellular traps. Down-regulation of ELANE can suggest that the influence of NETs on biocalcification is reduced [77]. The high frequency of up-regulated immunoglobulin mu heavy chain (P0DOX6) clearly shows the activity of the immune system against the present bacteria.

#### LIP sialoliths

For all samples from the LIP group, 84 statistically significant proteins were quantified in total samples from the LIP group. Most of the proteins were down-regulated for this group's samples (Additional file 1: Table S3). The next step was selecting the set of statistically significant up-regulated and down-regulated proteins detected in more than 50% of samples from the LIP group. Heatmap for these 22 proteins presents the level of regulation of proteins (Fig. 7B). In the group of unique proteins for the lipid type of salivary stones, there are 4 quantified proteins. These proteins were marked with yellow circles on the STRING-generated network (Fig. 6B). The group of LIP unique proteins includes transthyretin (TTR), lactotransferrin (LTF), matrix Gla protein (MGP) and submaxillary gland androgen-regulated protein 3A (SMR3A). Transthyretin and submaxillary gland androgen-regulated protein 3A were down-regulated in all lipid samples. Lactotransferrin and matrix Gla protein were up-regulated in only one LIP20 sample but down-regulated in the others. This sample in the PCA analysis deviated from the group and was in the range of mixed stones (Additional file 1: Figure S5).

Transthyretin (TTR) can form oligomers, which are responsible for increasing the concentration of calcium ions in cells, so down-regulation of TTR can be caused a lower level of calcium in lipid salivary stones [78]. There is a similar case with matrix Gla protein (MGP)—this protein has a high affinity to calcium ions, so decreasing the level of MGP can influence calcium balance [79]. Another example is lactotransferrin (LTF), an antimicrobial protein whose activity depends on the level of extracellular cations. It was assumed that in lipid sialoliths, the concentration of calcium ions is lower, and the down-regulation of this protein in most samples causes antimicrobial activity inhibition. Submaxillary gland androgen-regulated protein 3A (SMR3A) is secreted only by submaxillary glands into saliva, so its decreased level in all samples can be evidence of activity and functions of salivary glands are disturbed [80]. Non-unique but down-regulated for a significant part of all of the lipid samples is the eosinophil cationic protein (RNASE3), mentioned earlier, responsible for defence activity against the pathogens. Azurocidin 1 (AZU1), also known as cationic antimicrobial protein CAP37 or heparin-binding protein, is an important multifunctional inflammatory mediator, and its activity is mainly directed against Gram-negative bacteria [81]. Azurocidin 1 has inhibitory activity during periodontitis, so its variable level can cause different pathological states [82]. The activating function of azurocidin 1 concerning the macrophages is connected to the immobilisation of calcium ions, which is decreased in this group of sialoliths. However, in consequence, the concentration of Ca^2+^ in cells of salivary gland tissue can be higher, and that imbalance causes different dysfunctions [83]. This protein is also part of NETs [84]. Focusing on the different types of regulation of immunoglobulin alpha-2 heavy chain (P0DOX2), the body’s response to pathological bacteria is variable. Statherin (STATH) was mentioned earlier as a protein responsible for stabilising saliva supersaturated with calcium salts by inhibiting the precipitation of calcium phosphate salts. A variable level of STATH can indicate calcium imbalance in lipid stones.

#### MIX sialoliths

For all samples from the MIX group, 114 statistically significant proteins were quantified. In this case, the type of regulation of proteins is more varied in this group (Additional file 1: Table S4). The set of statistically significant up-regulated and down-regulated proteins detected in more than 50% of samples from the MIX group composed of 26 proteins is presented as a heatmap for this set presents the level of regulation of proteins (Fig. 7C). In the group of unique proteins for the mixed type of salivary stones, there was only 1 quantified protein—fibrinogen alpha chain (FGA). This protein is marked with a yellow circle on the STRING-generated network (Fig. 6C). FGA was down-regulated for all of the samples in the group. Fibrinogen is allowed to protect the neutrophils against the cytotoxic effects caused by, for example, the presence of bacteria and, consequently, the formation of neutrophil extracellular traps is delayed. The reduced level of fibrinogen alpha chain can cause the abnormal activity of neutrophils, leading to the formation of NETs [89, 90]. Non-unique but down-regulated in many samples was eosinophil cationic protein (RNASE3) and immunoglobulin gamma-1 heavy chain (P0DOX5), pointing to the disturbance in the immune response. Other non-unique frequent proteins have a different level of regulation among the MIX samples. These include, for example, haemoglobin subunit beta (HBB), which can harm oral mucosa because of the presence of the reactive heme group [85]. Cathepsin G (CTSG) and leukocyte elastase inhibitor (SERPINB1) are engaged in the immune response of the body against pathogens [86]. Zinc-alph-2-glycoprotin (AZGP1) is responsible for the degradation of lipids [87], but on the other hand, carbonic anhydrase (CA1) is a protein present in saliva, and its primary function is controlling the process of calcification [88].

## Discussion

Although the salivary stone disease is a common pathological state, it can cause serious consequences, such as severe pain, discomfort, nerve injuries and the necessity of re-surgical operation to remove sialoliths. Despite many theories, there is no confirmed cause of pathogenesis leading to salivary stone formation. Thanks to the use of qualitative, quantitative and bioinformatic analysis of MS data, we were able to examine protein compositions of sialoliths collected from patients taking into account their classification to calcified, lipid and mixed groups based on spectroscopic methods. Using advanced software for proteomic analysis, we had the chance to identify human and bacterial proteins.

### Bacterial proteins

Thanks to the Multi-round search option in PeaksSTUDIO software, raw spectra were searched first against the human and next against the bacterial database. Detected bacterial proteins belonged to 44 bacteria species. The most numerous bacteria group for which the proteins were identified was *Actinomyces*, which belongs to gram-positive opportunistic pathogens common in the oral cavity, especially in the gums, causing various oral infections [91]. Many *Actinomyces* species can cause actinomycosis associated with swelling and the formation of abscesses [92]. *Actinomyces viscosus* is a pathobiont which can colonize the oral cavity of even 70% of adults [93]. Its presence is connected with periodontal disease – *A. viscosus* was isolated from root surface caries and dental calculus [94]. *A. naeslundii* can also cause periodontal disease and is one of the first bacteria which can colonize the oral cavity and then cover the surface of teeth [95, 96]. *A. radicidentis* was also identified in infected root canals of teeth [97]. It is also part of the biofilm found on oral surfaces [98]. *Capnocytophaga sp. oral* is a gram-negative opportunistic pathogen involved in pathogenesis leading to periodontal disease [99]. It can often be isolated from periodontal pockets and abscesses [100]. The following identified bacteria was *Eikenella corrodens*, a Gram-negative bacterium common in the oral cavity but can act as an opportunistic pathogen [101]. Its presence can cause the formation of abscesses, including the area of submandibular glands [102]. Another large group of detected bacteria is *Fusobacterium*. According to the current statement, gram-negative *Fusobacterium* species should be permanently treated as pathogens—they cause several human diseases, including periodontal disease [91, 103]. The species responsible for that pathological state are, for example, *F. nucleatum* and *F. polymorphum*. Moreover, they can form aggregates with other bacteria in the oral cavity [104, 105]. Usually, the Gram-negative *Hamephilus* species are commensal organisms, including the mouth. They are also part of the salivary microbiome [106, 107]. However, detected *H. haemolyticus* and *H. parainfluenzae* are opportunistic pathogens whose presence leads to the formation of abscesses [108]. 4 species from *Neisseria* group were detected in sialoliths: *N. bacilliformis*, *N. macacae*, *N. sicca* and *N. sp. oral*. These Gram-negative bacteria can be found on mucosal surfaces.

In most cases, they are commensals but living in the oral cavity, and they can act as opportunistic pathogens, causing some diseases and infections [109, 110]. *Porphyromonas sp. oral* is gram-negative bacteria commonly present in the oral cavity and found in the salivary microbiome [111, 112]. As pathobiont in the case of disturbed homeostasis, *Porphyromonas* can cause different diseases, for example, periodontitis [113, 114]. *Pseudopropionibacterium propionicum,* a Gram-positive bacteria and opportunistic pathogen, form biofilm on external root surfaces of teeth, may cause endodontic pathological states and leads to actinomycosis [115–117]. Gram-positive *Rothia dentocariosa* is a standard part of the oral and respiratory tract microbiome [118]. According to one of the hypotheses, this bacterium was associated with periodontal disease. This pathological state caused by *R. dentocariosa* can lead to infections of other tissues [119]. They identified proteins from Gram-negative *Tannerella forsythia* and *Treponema denticola* in clinical sialolith samples and *Porphyromonas gingivalis* (not detected in this research but shown in other) from the Red Complex. They are the primary virulent pathogens which cause chronic periodontitis [120]. Other species of bacteria were also detected based on the analyzed proteins, usually belonging to the natural microbiome of the oral cavity. However, they transformed into pathogens under stressful conditions and were allowed to cause variable diseases. They were primarily Gram-negative species: *Aggregatibacter aphrophilus*, *Desulfobulbus oralis*, *Fretibacterium sp.*, *Kingella potus*, *Ottowia sp. oral*, *Selenomonas noxia* and *Selenomonas sp. oral*. There were also Gram-positive species: *Peptostreptococcus stomatis* and *Streptococcus mitis*. As we can see, bacteria are commonly present in the oral cavity (gums, saliva, surface of teeth), and often, they are causes of periodontal diseases. It can also suggest their potential influence on pathogenesis leading to the development of salivary stone disease.

Some of the identified bacteria species, for which the proteins were extracted and detected from clinical samples of salivary stones, were also shown in previous research describing the presence of bacterial pathogens in sialoliths and their potential influence on pathogenesis and mineralisation leading to the formation of deposits in salivary glands. There were detected precisely the same species: *Actinomyces viscosus*, *Eikenella corrodens*, *Fusobacterium nucleatum*, *Haemophilus parainfluenzae* and *Streptococcus mitis*. What is more, identified bacteria, if they were not the same species, they were from the same groups: *Actinomyces*, *Capnocytophaga*, *Eikenella*, *Haemophylus*, *Kingella*, *Neisseria*, *Peptostreptococcus*, *Porphyromonas*, *Rothia* and *Streptococcus* [22, 42, 46–48]. All of the identified bacteria groups are common in the oral cavity. In most cases, they are opportunistic pathogens which can cause various infections and diseases when the environmental homeostasis is disturbed. There were also selected bacteria species that occur uniquely in different types of sialoliths and are standard for all 3 salivary stones (Table 2). These sets are numerous, taking into account limited samples in each group. A similar analysis should be conducted using more clinical samples to confirm the uniqueness and repeatability of these species. Besides, the protein extraction protocol should be improved to obtain a more significant number of proteins, especially bacterial proteins.

### Human proteins

Qualitative analysis of identified human proteins in the studied groups of sialoliths, supported by enrichment and functional analysis, showed that the most often repeated proteins between groups with the highest frequency are- neutrophil defensin 3 (DEFA3), protein S100A9 and S100-A8, cathepsin G (CTSG), myeloperoxidase (MPO), lactotransferrin (LTF), eosinophil cationic protein (RNASE3) and lysozyme C (LYZ)—according to the GO enrichment mainly involved in the defence response to the bacterium, regulated exocytosis and neutrophil degranulation. Most of the proteins identified and shown in Fig. 4 are multifunctional, suggesting a complex process influenced by many factors. Neutrophil defensin 3 has antibiotic, fungicide and antiviral activities. The group of neutrophil defensins can kill microorganisms by permeabilizing their plasma membrane [121] and are present in the granules of neutrophils and the epithelia of mucosal surfaces such as the oral cavity [122]. The decreased level of neutrophil defensin 3 was noted in the case of dental caries. It is connected with antimicrobial activity because, in the pathogenesis of periodontitis, antimicrobial proteins such as DEFA3 can cooperate with other inflammatory proteins and regulate distinct inflammatory pathways [123, 124]. Neutrophil defensin 3 is also associated with dyslipidemia causing lipids imbalance and influencing the lipid and calcium balance in sialolithiasis [125]. On the other hand, proteins S100A8 and S100A9 are essential calcium- and zinc-binding proteins and form a complex called calprotectin [126], which has antimicrobial properties thanks to the metal sequestration process conducted in the presence of calcium by chelation [127–129]. A low level of S100-A9 is the cause of neutrophil extracellular trap formation (NETs) in the presence of bacteria [130]. The formation of these structures directly influences calcification, leading to sialolith generation. On the other hand, increased level of proteins S100-A8 and S100-A9 is treated as a marker of periodontitis [131], indicating that an imbalance of these 2 proteins can cause oral pathological states. Cathepsin G (CTSG) is a protein which also plays antibacterial activity. Moreover, this property can also allow fighting against biofilms, which can play an essential role in sialolith formation [132–134]. Cathepsin G belongs to the neutrophil serine proteases family. This protein was first identified as a degradative enzyme that acts at inflammatory sites in 2 ways—intracellularly (degradation of pathogens) and extracellularly (breakdown of extracellular matrix components) [135]. CTSG is also localized in NETs – essential for forming sialoliths—because of its high affinity for chromatin [77]. This protein causes changes in the level of calcium ions during exposure to endothelial cells [136]. On the other hand, the cathepsin family was also detected as a mediator of the metabolism of lipids [137], so an imbalance of lipids and calcium can also be caused by the level disturbance of cathepsin G. Myeloperoxidase is a peroxidase enzyme expressed primarily on neutrophil granulocytes. Their antimicrobial activity is connected with the secretion of hypohalous acids [138, 139]. Increased level of MPO was noted in saliva in patients with periodontitis [140]. Focusing on the balance of calcium and lipid, first, myeloperoxidase has a high affinity for calcium [141]. Second, in the presence of pathogenic bacteria, MPO initiates lipid peroxidation during the inflammation process, changing their features [142]. Myeloperoxidase can also be found in NETs [84]. Lactotransferrin (LTF) is a multifunctional protein of the transferrin family and has antimicrobial activity. This feature depends on the extracellular cation concentration [143], possibly connected with the calcium ions' level. LTF is present mainly in secretory fluids, such as saliva [144]. Lactoferrin in saliva decreases bacterial growth, biofilm development and inflammatory processes [145, 146]. LTS is also a biomarker of salivary gland pathological states [147]. This protein also takes part in the creation of NETs [84]. Eosinophil cationic protein (RNASE3) is a heparin-binding ribonuclease with cytotoxic abilities [148]. This feature is used against many pathogens – lipid bilayers of pathogenic microorganisms are destabilized [149]. The concentration of eosinophil cationic protein is increased in plasma and other body fluids, also in saliva, during inflammation [150]. There are many studies about the role of eosinophil cationic protein as a biomarker of asthma [151]. Lysozyme (LYZ) is another antimicrobial enzymatic protein. It causes hydrolyzation of peptidoglycan—the crucial component of the cell walls of Gram-positive bacteria. Thanks to that, the bacterial cells are lysed [152]. Moreover, lysozyme and calcium cause an imbalance of calcium concentration [153]. Besides, LYZ is also part of neutrophil extracellular traps [84].

### Quantitative analysis

#### Calcified sialoliths

Analysing Biological Process GO terms (Additional file 1: Figure S2), most of them are connected with the body’s defence against bacteria, which proteins were identified earlier. About 50% of analysing proteins are involved in these processes and up-regulated for all calcified samples. Only 4 proteins: neutrophil elastase (ELANE), statherin (STATH), eosinophil cationic protein (RNASE3) and azurocidin (AZU1) are down-regulated for all of the samples. Other 4 proteins: immunoglobulin J chain (IGJ), myeloperoxidase (MPO), cathepsin G (CTSG), and immunoglobulin lambda constant 2 (P0DOY2), have varied values of fold change among the samples in the calcified group. These changes are directly connected with pathological states caused by pathogenic bacteria.

Focussing on the Cellular Component GO terms, all of them are associated with creating extracellular components, referring to the hypothesis about the role of neutrophil extracellular traps in the biocalcification leading to the sialoliths formation. In this case, only keratin 4 (KRT4) is not involved.

Most of the proteins are up-regulated for all of the samples. 7 proteins: neutrophil elastase (ELANE), statherin (STATH), submaxillary gland androgen-regulated protein 3B (SMR3B), eosinophil cationic protein (RNASE3), azurocidin (AZU1), haemoglobin subunit beta (HBB) and neutrophil gelatinase-associated lipocalin (LCN2), which are involved in the formation of cellular components, are down-regulated. Other 6 proteins: carbonic anhydrase 1 (CA1), immunoglobulin J chain (IGJ), myeloperoxidase (MPO), cathepsin G (CTSG), immunoglobulin lambda constant 2 (P0DOY2) and alpha-amylase 1A (P0DUB6) have a different level of regulation among the samples in the group. For Biological Process and Cellular Component GO terms, the values of -log(q-value) are high, pointing to the high statistical significance of the identified terms. However, it can be caused by numerous groups of analysing proteins. Detected Molecular Function GO terms are mostly connected to antimicrobial activities, but fewer proteins were linked to these terms compared to previous results. One of the most important conclusions is detecting the *Neutrophil extracellular trap formation* KEGG pathway based on quantified proteins. It is evidence of the crucial role of NETs in salivary stones formation, but the group of proteins involved in this process is relatively small. Several proteins also influence the *Salivary secretion* KEGG pathway, so modifying this process may have an essential role in pathogenesis. What is more, identified *Neutrophil degranulation* Reactome pathway can be indicative of the ongoing process of NETs formation because, during the degranulation of neutrophils, their chromatin is released. Most of the proteins associated with the *Neutrophil degranulation* Reactome pathway are up-regulated, so we suppose this process intensifies.

#### Lipid sialoliths

Focusing on Biological Process GO terms (Additional file 1: Figure S3), about half of the proteins of LIP sialoliths are associated with the body’s defence against bacteria, so this part of selected proteins is smaller than in the case of the CAL group. Most of them have a variable level of regulation among the sample in the group. Only 2 proteins, eosinophil cationic protein (RNASE3) and myeloperoxidase (MPO), are down-regulated for all of the samples in the group. This imbalance directly shows the influence of bacteria on the pathological state leading to the formation of sialoliths. However, comparing this analysis with results for calcified stones, it is suggested that the body’s response against the pathogens is at a lower level. Several proteins were connected with the body’s activity against fungus, so there is the possibility that oral fungal infections can influence the development of salivary stone disease, but similar terms were not connected with proteins selected for calcified stones. Some of the proteins are responsible for the regulation of endopeptidase activity. Most often, these proteins have a variable level of regulation, 3 proteins: submaxillary gland androgen-regulated protein 3B (SMR3B), leukocyte elastase inhibitor (SERPINB1), submaxillary gland androgen-regulated protein 3A (SMR3A) are down-regulated among the whole group. Endopeptidase activity is associated with lipids, so disturbing levels of these proteins can result from the imbalance of lipids [154]. These functions were not detected in the case of calcified sialoliths. Detected Cellular Component GO terms show the connection between the formation of salivary stones and neutrophil extracellular matrix. All of the LIP group's proteins are involved in this process. They usually have a variable type of regulation, but 5 proteins: eosinophil cationic protein (RNASE3), transthyretin (TTR), submaxillary gland androgen-regulated protein 3B (SMR3B), myeloperoxidase (MPO) and leukocyte elastase inhibitor (SERPINB1) are down-regulated for all of the samples. Analysing Molecular Function GO terms, several proteins are again involved in peptidases and endopeptidases activity. 3 of these proteins are down-regulated, the same as those detected for Biological Process GO. Again, the *Neutrophil extracellular trap formation* KEGG pathway and *Neutrophil degranulation* Reactome pathway were detected, confirming the pivotal role of NETs in biocalcification leading to the salivary stones formation. The values of − log(q-value) are lower, but it is probably the result of the less numerous group of analysing proteins.

#### Mixed sialoliths

Analysing Biological Process GO terms (Additional file 1: Figure S4), about half of the proteins are associated with the body’s defence against bacteria, so this part of selected proteins is smaller than in the case of the CAL group but roughly equal to the part of the LIP group. Most of them have a variable level of regulation among the sample in the group. Only 4 proteins, eosinophil cationic protein (RNASE3), azurocidin (AZU1), fibrinogen alpha chain (FGA) and neutrophil elastase (ELANE), are down-regulated for all of the samples in the group. Again, as in the case of lipid sialoliths, we can suppose that the body’s response against the pathogens is lower than calcified salivary stones. Terms describing the body’s activity against fungus were not detected in this case. Some proteins are responsible for the regulation of endopeptidase activity. However, this group of proteins is less numerous, and the values of -log(q-value) are lower than in the enrichment analysis of the LIP group. The Biological Processes connected with the regulation of endopeptidase activity were not identified for MIX sialoliths. Cellular Component GO terms indicate the role of neutrophil extracellular matrix in the sialolithiasis, and analysing the values of -log(q-value), these terms have higher significance than in the case of the lipid group but lower than the calcified group. Most of the MIX group proteins are involved in these terms. They usually have a variable type of regulation, but 5 proteins: eosinophil cationic protein (RNASE3), azurocidin (AZU1), fibrinogen alpha chain (FGA), submaxillary gland androgen-regulated protein 3B (SMR3B) and neutrophil elastase (ELANE) are down-regulated for all of the samples. About Molecular Function GO terms, fewer proteins are involved in peptidases and endopeptidase activity compared to the lipid sialoliths. The significance is also on the lower level. *Neutrophil extracellular trap formation* and *Salivary secretion* KEGG pathways and *Neutrophil degranulation* Reactome pathway were detected again. Proteins connected with these 2 terms have mainly variable levels of regulation.

#### Comparison of CAL, LIP and MIX sialoliths

The unique protein for the calcified and lipid group was immunoglobulin lambda-like polypeptide 5 (IGLL5), engaged in the immune system response. In all of the calcified samples for which this protein was quantified (5 of 6), its level was up-regulated, and the level of its regulation in lipid samples was varied—up-regulated in 1 sample and down-regulated in 2 samples. The unique proteins for calcified and mixed sialolith groups included 8 proteins. Carbonic anhydrase 1 (CA1), a protein responsible for calcification, has a variable type of regulation for both CAL and MIX groups, pointing to the imbalance of calcium. Up-regulated immunoglobulin mu heavy chain (P0DOX6) and variable immunoglobulin lambda constant 2 (P0DOY2) are evidence of the immune system’s activity caused by bacteria. Albumin (ALB) can bind calcium ions, so the up-regulation of this protein in the CAL group and variable level in the MIX group is completely understandable [155]. The same types of regulation as for albumin were detected for mentioned earlier keratin, type I cytoskeletal 19 (KRT19). This protein-fixing structural balance can change the metabolism of lipids. Serotransferrin (TF) with the same type of regulation belongs to the transferrin family typical in saliva. Hence, its activity depends on the concentration of ions and calcium ions, which is crucial in calcified sialoliths, where TF is up-regulated. Alpha-amylase 1A (P0DUB6), common in saliva, is a calcium-binding protein, so its variable level in salivary stones indicates calcium imbalance [156]. Neutrophil gelatinase-associated lipocalin (LCN2) takes part in the regulation of immune response in the presence of bacteria, and as a member of the lipocalin family, this protein is responsible for the transport of hydrophobic molecules, such as lipids. Down-regulation of LCN2 can cause lipids deficiency in calcified salivary stones. Besides, LCN2 is also part of NETs [84].

On the other hand, the set of unique proteins for lipid and mixed sialolith groups included 4 proteins. Variable levels of protein S100-A9 (S100A9) and zinc-alpha-2-glycoprotein (AZGP1) can influence the balance of calcium and lipids, respectively. Besides, S100A9 modifies the formation of NETs. Haemoglobin subunit alpha (HBA2), such as another described earlier subunit, haemoglobin subunit beta (HBB), can cause damage to the oral mucosa, accelerating inflammation. Leukocyte elastase inhibitor (SERPINB1) and its down-regulation in the LIP group and variable level in the MIX group show that the body’s response to the presence of bacteria is clearly at a lower level in the case of lipid sialoliths. The unique proteins for these 2 pairs of sialolith groups are present in Additional file 1: Table S5, considering the regulation type.

The Standard set of proteins for each type of salivary stone included 13 proteins: azurocidin (AZU1), cystatin-SN (CST1), cystatin-S (CST4), cathepsin G (CTSG), neutrophil elastase (ELANE), haemoglobin subunit beta (HBB), histone H3,1 (HIST1H3J), myeloperoxidase (MPO), eosinophil cationic protein (RNASE3), submaxillary gland androgen-regulated protein 3B (SMR3B), statherin (STATH), immunoglobulin alpha-2 heavy chain (P0DOX2), immunoglobulin gamma-1 heavy chain (P0DOX5). The level of regulation of each protein for all samples is presented as a heatmap (Additional file 1: Figure S6).

For the standard statistically significant proteins quantified in more than 50% of samples in each group, the STRING-generated network presenting experimentally verified protein–protein interactions was prepared (Additional file 1: Figure S7). The network presents the type of regulation of proteins in each group and their frequency among the samples. Most of the proteins were mentioned above. The activity of azurocidin (AZU1) concerning the role of macrophages during the body's immune response depends on calcium ions inside the cells. Down-regulation of AZU1 in the case of calcium and mixed sialoliths can be associated with immobilising a more significant amount of calcium in stones, thus reducing intracellular Ca^2+^. Without a proper amount of calcium ions, azurocidin cannot fulfil its functions, even in the presence of pathogens. The role of azurocidin in the creation of NETs is also reduced. A disturbed calcium balance in lipid stones leads to a variable level of AZU1 in these sialoliths. Myeloperoxidase (MPO) has a high affinity for calcium, so different concentrations of MPO in CAL and MIX groups point to the imbalance of this mineral compound in salivary stones and the presence of bacteria. Down-regulation of MPO in the case of lipid stones shows the lower concentration of calcium in these sialoliths and immobilisation of lipids, so their peroxidation is reduced, reduced role of this protein during NETosis and the smaller number of identified bacteria in lipid sialoliths. Neutrophils secrete neutrophil elastase (ELANE) during inflammation, so down-regulation of this protein in calcified and mixed groups suggests a lower level of immune response mediated by this protein and reduced part of ELANE in the neutrophil extracellular trap. Cathepsin G (CTSG), as a mediator of metabolisms of calcium and lipids, quantified in all of the groups on the different levels, can cause calcium-lipid imbalance and influence the formation of NETs. At the same time, its presence clearly shows the influence of bacteria on the calcification process. Haemoglobin subunit beta (HBB) is down-regulated for calcified and lipid sialoliths, suggesting that the risk of harming oral mucosa is reduced. The role of statherin (STATH) is the protection of saliva from the precipitation of calcium phosphate salts in high concentrations. Reduced levels of STATH in calcified stones can be associated with lower calcium salts in saliva concentration caused by the immobilization of calcium in sialoliths. Submaxillary gland androgen-regulated protein 3B (SMR3B) is a poorly described protein, but there is a prediction that SMR3B can inhibit the activity of peptidases and endopeptidases, which, as it was mentioned above, are connected with the presence of lipid [157]. Variable types of regulation of this protein in LIP and MIX stones indicate some imbalance of this mineral compound in these stones when the lipid concentration is higher in the LIP group, inhibiting peptidases and endopeptidases can be difficult. This way, the lowest level of submaxillary gland androgen-regulated protein 3B in lipid sialoliths is explained. Cystatin-SN (CST1) and cystatin-S (CST4) are similar proteins common in saliva. Their primary function is the inhibition of human cathepsins, which is why the level of cathepsin G (CTSG) is variable [50]. Cystatins S and SN can bind calcium, so their up-regulation in CAL sialoliths show a high concentration of this mineral compound. Different level in LIP and MIX salivary stones probably causes an imbalance of calcium in these groups. Histones are nuclear proteins that tightly pack DNA into chromatin [158]. During NETosis, chromatin is decondensed, and the level of decondensation can be regulated by histone H3,1 (HIST1H3J) [159]. Variable types of regulation of HIST1H3J in all groups may influence chromatin decondensation, leading to the creation of neutrophil extracellular matrix and calcification. Eosinophil cationic protein (RNASE3), immunoglobulin alpha-2 heavy chain (P0DOX2) and immunoglobulin gamma-1 heavy chain (P0DOX5) are mainly responsible for the body’s defence against pathogens, so down-regulation of eosinophil cationic protein and immunoglobulin gamma-1 heavy chain and variable level of immunoglobulin alpha-2 heavy chain shows, that the immune response in the presence of bacteria is disturbed. That effect can have a strong influence on the formation of sialoliths. 6 proteins have a common type of regulation comparing pairs CAL-MIX (azurocidin, myeloperoxidase, neutrophil elastase) and LIP-MIX (statherin, cystatin-SN, cystatin-S), proving that mixed sialoliths have features of both calcified and lipid stones. The following 6 proteins are down-regulated (eosinophil cationic protein, submaxillary gland androgen-regulated protein 3B, immunoglobulin gamma-1 heavy chain) or have variable levels (cathepsin G, immunoglobulin alpha-2 heavy chain, histone H3,1) for all of the groups. Haemoglobin subunit beta is regulated differently in the case of mixed salivary stones. (Additional file 2: Table. S6)

Biological Process GO terms (Additional file 1: Figure S8) detected for the common 13 proteins for all types of sialoliths are mainly associated with the body’s immune defence against pathogenic bacteria. 7 quantified proteins evidence it: azurocidin (AZU1), cystatin-S (CST4), cystatin-SN (CST1), submaxillary gland androgen-regulated protein 3B (SMR3B), myeloperoxidase (MPO), neutrophil elastase (ELANE) and cathepsin G (CTSG). In Cellular Component GO enrichment, detected terms are connected to extracellular components, pointing to the creation of neutrophil extracellular traps during calcification leading to the formation of sialoliths. There are 6 proteins responsible for the regulation of this process: statherin (STATH), azurocidin (AZU1), eosinophil cationic protein (RNASE3), myeloperoxidase (MPO), neutrophil elastase (ELANE) and cathepsin G (CTSG). Focusing on the Molecular Functional GO enrichment, summarizing all of the proteins are associated with modulation of peptidases and endopeptidases, which activity can be disturbed by the imbalance of lipids. Also, for this set of proteins, the *Neutrophil extracellular trap formation* KEGG pathway was detected, highlighting the role of NETosis in forming stones. In this pathway, there are engaged 5 proteins: azurocidin (AZU1), myeloperoxidase (MPO), neutrophil elastase (ELANE), cathepsin G (CTSG) and histone H3,1 (HIST1H3J). The influence on the *Salivary secretion* KEGG pathway has statherin (STATH), Cystatin-SN (CST1) and cystatin-S (CST4), and this can also have negative consequences. The Reactome pathways concern mainly immune response, but the most important for NETosis is *Neutrophil degranulation* Reactome pathway engaging 6 proteins: azurocidin (AZU1), eosinophil cationic protein (RNASE3), myeloperoxidase (MPO), neutrophil elastase (ELANE), cathepsin G (CTSG) and histone H3,1 (HIST1H3J).

## Conclusions

Work on the study of the composition and the search for biomarkers of all kinds of solid deposits formed in the human body is very important for understanding the processes leading to them. Although sialoliths are not the most accessible research material, it is possible to conduct qualitative and quantitative analyses of human proteins in their composition using proteomics. It is also possible to identify bacterial proteins, which requires further refinement. Using lysis buffers directed to the lysis of bacterial cells may increase the number of identifications or improve the results’ quality. The presented study analysed the protein compositions of salivary gland stones classified into three groups: calcified CAL, lipid LIP and mixed MIX. Classification based on spectroscopic methods should be supported by proteomic analysis, which verifies the classification of clinical samples in a good way. Qualitatively, common proteins in all these groups were found, and thanks to quantitative analysis, proteins unique to each of them were identified. Most such proteins were identified for the CAL type, and the MIX group was the least unique. The results also indicate that neutrophil extracellular trap formation (NETs) may be crucial for forming this type of deposit.

## Supplementary Information

**Additional file 1: Figure S1.** Chart presenting the numbers of human and bacterial proteins groups at 1% FDR with minimum 2 different peptides (bar graph) and the numbers of bacteria species from which the bacterial proteins were detected in each sample (blue curve). **Table S1.** All of the bacteria species, from which the proteins at 1% FDR with minimum 2 different peptides, were detected. **Table S2.** A table with the number of up-regulated and down-regulated proteins for each clinical sample from the CAL group, which are statistically significant (q<0.05) and the log2FC≥0.45 for up-regulated proteins and log2FC≤-0.45 for down-regulated proteins. **Table S3.** A table with the number of up-regulated and down-regulated proteins for each clinical sample from the LIP group, which are statistically significant (q<0.05) and the log2FC≥0.45 for up-regulated proteins and log2FC≤-0.45 for down-regulated proteins. **Table S4.** A table with the number of up-regulated and down-regulated proteins for each clinical sample from the MIX group, which are statistically significant (q<0.05) and the log2FC≥0.45 for up-regulated proteins and log2FC≤-0.45 for down-regulated proteins. **Figure S2.** Visualizing enrichment analysis of the most common proteins identified in the CAL group considers Biological Process GO, Cellular Component GO, Molecular Function GO, KEGG and Reactome terms with the highest significance (the highest values of -log(q-value)). The names of proteins marked with yellow colour point unique quantified proteins for the CAL group. Fill colours correspond with the type of regulation of protein: red—up-regulation of protein among the whole group, blue – down-regulation of protein, and green—level o regulation of protein is varied among the proteins in the group. **Figure S3.** Visualizing enrichment analysis of the most common proteins identified in the LIP group considers Biological Process GO, Cellular Component GO, Molecular Function GO, KEGG and Reactome terms with the highest significance (the highest values of − log(q-value)). The names of proteins marked with yellow colour point unique quantified proteins for the LIP group. Fill colours correspond with the type of regulation of protein: red - up-regulation of protein among the whole group, blue—down-regulation of protein, and green—level o regulation of protein is varied among the proteins in the group. **Figure S4.** Visualizing enrichment analysis of the most common proteins identified in the MIX group considers Biological Process GO, Cellular Component GO, Molecular Function GO, KEGG and Reactome terms with the highest significance (the highest values of -log(q-value)). The name of the protein marked with a yellow colour point is a unique quantified protein for the MIX group. Fill colours correspond with the type of regulation of protein: red - up-regulation of protein among the whole group, blue—down-regulation of protein, and green—level o regulation of protein is varied among the proteins in the group. **Figure S5.** PCA analysis of all the clinical sialolith and pooled samples were used in relative quantitative analysis. Data were normalised using the total area sums (TAS) approach, and technical replicates of samples were averaged. **Table S5.** A table presents the level of regulation of proteins, which is statistically significant (q<0,05) and the log2FC≥0,45 for up-regulated proteins and log2FC≤-0,45 for down-regulated proteins for more than 50% of proteins from each group and are typical for paired sialolith groups. Red colour corresponds with the up-regulation of protein among the whole group, blue—is the down-regulation of protein, and green—level o regulation of protein regulation varies among the group proteins. **Figure S6.** Heatmap presents the level of regulation of proteins, which are statistically significant (q<0,05) and the log2FC≥0,45 for up-regulated proteins and log2FC≤-0,45 for down-regulated proteins for more than 50% of samples standard for CAL, LIP and MIX groups. The red colour corresponds with values of log(FC) for up-regulated proteins, and the blue corresponds with values of log(FC) for down-regulated proteins. **Figure S7.** The Cytoscape visualisation of the STRING-generated network is composed of experimentally verified protein-protein interactions among the proteins common for CAL, LIP and MIX groups. The size of nodes corresponds with the frequency of occurrence of protein among all of the samples in the group; bigger nodes represent higher frequency, and smaller—ones represent lower frequency. Fill colours correspond with the type of regulation of protein: red—up-regulation of protein among the whole group, blue—down-regulation of protein, and green—level of regulation of protein is varied among the proteins in the group. **Figure S8.** The visualization of enrichment analysis of the proteins common for CAL, LIP and MIX groups. Fill colours correspond with the type of regulation of protein: red—up-regulation of protein among the whole group, blue—down-regulation of protein, and green—level o regulation of protein is varied among the proteins in the group.

**Additional file 2.** Statistical analysis of data obtained from the SWATH-MS analysis for the studied sialoliths. The table contains pool samples and each analyzed stone with values such as: t-test value and p-value, q-value, medians, fold change and log2 fold change, which were used to type statistically significant changes in protein levels.

## Data Availability

The mass spectrometry proteomics data have been deposited to the ProteomeXchange Consortium via the PRIDE partner repository with the dataset identifier PXD039381.
