# Supplementary material for: Proteomic analysis of sialoliths from calcified, lipid and mixed groups as a source of potential biomarkers of deposit formation in the salivary glands
Source: Clin Proteomics. 2023 Mar 22;20:11. doi: 10.1186/s12014-023-09402-3 (PMC10035263; doi:10.1186/s12014-023-09402-3)

**Additional file document**

Natalia Musiał^1*^, Aleksandra Bogucka^1,2^, Dimitry Tretiakow^3^, Andrzej Skorek^3^, Jacek Ryl^4^,

Paulina Czaplewska^1*^

^1^ Intercollegiate Faculty of Biotechnology UG&MUG, University of Gdańsk, Abrahama 58, 80-307 Gdańsk, Poland

^2^ Institute of Biochemistry, Medical Faculty, Justus Liebig University of Giessen, Friedrichstrasse 24, 35392 Giessen, Germany
^3^ Department of Otolaryngology, Faculty of Medicine, Medical University of Gdańsk, Smoluchowskiego 17, 80-214 Gdańsk, Poland

^4^ Division of Electrochemistry and Surface Physical Chemistry, Faculty of Applied Physics and Mathematics, Gdańsk University of Technology, G. Narutowicza 11/12, 80-233 Gdańsk, Poland

***Corresponding authors**:

Paulina Czaplewska, PhD. Intercollegiate Faculty of Biotechnology UG&MUG, University of Gdańsk, Abrahama 58, 80-307 Gdańsk, Poland. [paulina.czaplewska@ug.edu.pl](mailto:paulina.czaplewska@ug.edu.pl), ORCID 0000-0002-6469-6014

Natalia Musiał, MSc, Intercollegiate Faculty of Biotechnology UG&MUG, University of Gdańsk, Abrahama 58, 80-307 Gdańsk, Poland. [natalia.musial@phdstud.ug.edu.pl](mailto:natalia.musial@phdstud.ug.edu.pl), ORCID 0000-0001-9883-7965

**Additional file 1: Figure S1.** Chart presenting the numbers of human and bacterial proteins groups at 1% FDR with minimum 2 different peptides (bar graph) and the numbers of bacteria species from which the bacterial proteins were detected in each sample (blue curve).


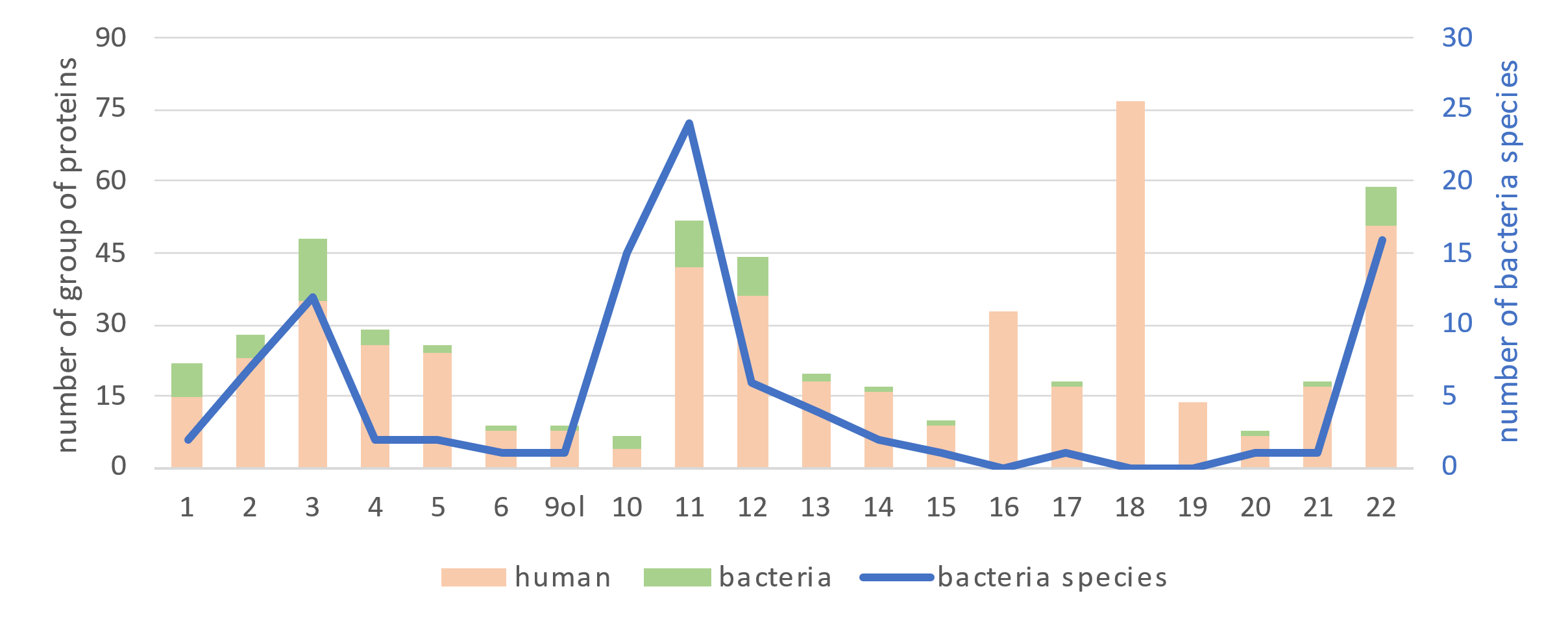


**Additional file 1: Table S1.** All of the bacteria species, from which the proteins at 1% FDR with minimum 2 different peptides were detected.

| *Actinomyces bowdenii* | *Actinomyces sp. Oral* | *Fretibacterium sp.* | *Haemophilus parainfluenzae* | *Porphyromonas sp. Oral* |
| --- | --- | --- | --- | --- |
| *Actinomyces glycerinitolerans* | *Actinomyces succiniciruminis* | *Fusobacterium canifelinum* | *Kingella potus* | *Pseudopropionibacterium propionicum* |
| *Actinomyces johnsonii* | *Actinomyces urogenitalis* | *Fusobacterium hwasookii* | *Neisseria bacilliformis* | *Rothia dentocariosa* |
| *Actinomyces massiliensis* | *Actinomyces viscosus* | *Fusobacterium nucleatum* | *Neisseria macacae* | *Selenomonas noxia* |
| *Actinomyces naeslundii* | *Aggregatibacter aphrophilus* | *Fusobacterium nucleatum subsp. Animalis* | *Neisseria sicca* | *Selenomonas sp. Oral* |
| *Actinomyces oris* | *Capnocytophaga sp. Oral* | *Fusobacterium nucleatum subsp. Polymorphum* | *Neisseria sp. Oral* | *Streptococcus mitis* |
| *Actinomyces radicidentis* | *Comamonadaceae bacterium* | *Fusobacterium periodonticum* | *Nitrospina sp.* | *Tannerella forsythia* |
| *Actinomyces ruminicola* | *Desulfobulbus oralis* | *Fusobacterium pseudoperiodonticum* | *Ottowia sp. Oral* | *Treponema denticola* |
| *Actinomyces slackii* | *Eikenella corrodens* | *Haemophilus haemolyticus* | *Peptostreptococcus stomatis* |  |

**Additional file 1: Table S2.** A table with the number of up-regulated and down-regulated proteins for each clinical sample from the CAL group, which are statistically significant (q<0.05) and the log2FC≥0.45 for up-regulated proteins and log2FC≤-0.45 for down-regulated proteins.

|  | sample 1 | sample 4 | sample 5 | sample 9 (outer layers) | sample 10 | sample 12 |
| --- | --- | --- | --- | --- | --- | --- |
| Number of up-regulated proteins | 18 | 89 | 41 | 30 | 93 | 48 |
| Number of down-regulated proteins | 6 | 15 | 8 | 7 | 11 | 17 |

**Additional file 1: Table S3.** A table with the number of up-regulated and down-regulated proteins for each clinical sample from the LIP group, which are statistically significant (q<0.05) and the log2FC≥0.45 for up-regulated proteins and log2FC≤-0.45 for down-regulated proteins.

|  | sample 3 | sample 11 | sample 19 | sample 20 |
| --- | --- | --- | --- | --- |
| Number of up-regulated proteins | 15 | 15 | 6 | 5 |
| Number of down-regulated proteins | 26 | 49 | 9 | 24 |

**Additional file 1: Table S4.** A table with the number of up-regulated and down-regulated proteins for each clinical sample from the MIX group, which are statistically significant (q<0.05) and the log2FC≥0.45 for up-regulated proteins and log2FC≤-0.45 for down-regulated proteins.

|  | sample 2 | sample 6 | sample 13 | sample 14 | sample 15 | sample 16 | sample 17 | sample 18 | sample 21 | sample 22 |
| --- | --- | --- | --- | --- | --- | --- | --- | --- | --- | --- |
| Number of up-regulated proteins | 76 | 4 | 15 | 25 | 2 | 20 | 5 | 37 | 5 | 18 |
| Number of down-regulated proteins | 14 | 20 | 14 | 11 | 19 | 13 | 19 | 29 | 70 | 46 |

**Additional file 1: Figure S2.** Visualizing enrichment analysis of the most common proteins identified in the CAL group considers Biological Process GO, Cellular Component GO, Molecular Function GO, KEGG and Reactome terms with the highest significance (the highest values of -log(q-value)). The names of proteins marked with yellow colour point unique quantified proteins for the CAL group. Fill colours correspond with the type of regulation of protein: red - up-regulation of protein among the whole group, blue – down-regulation of protein, and green – level o regulation of protein is varied among the proteins in the group.


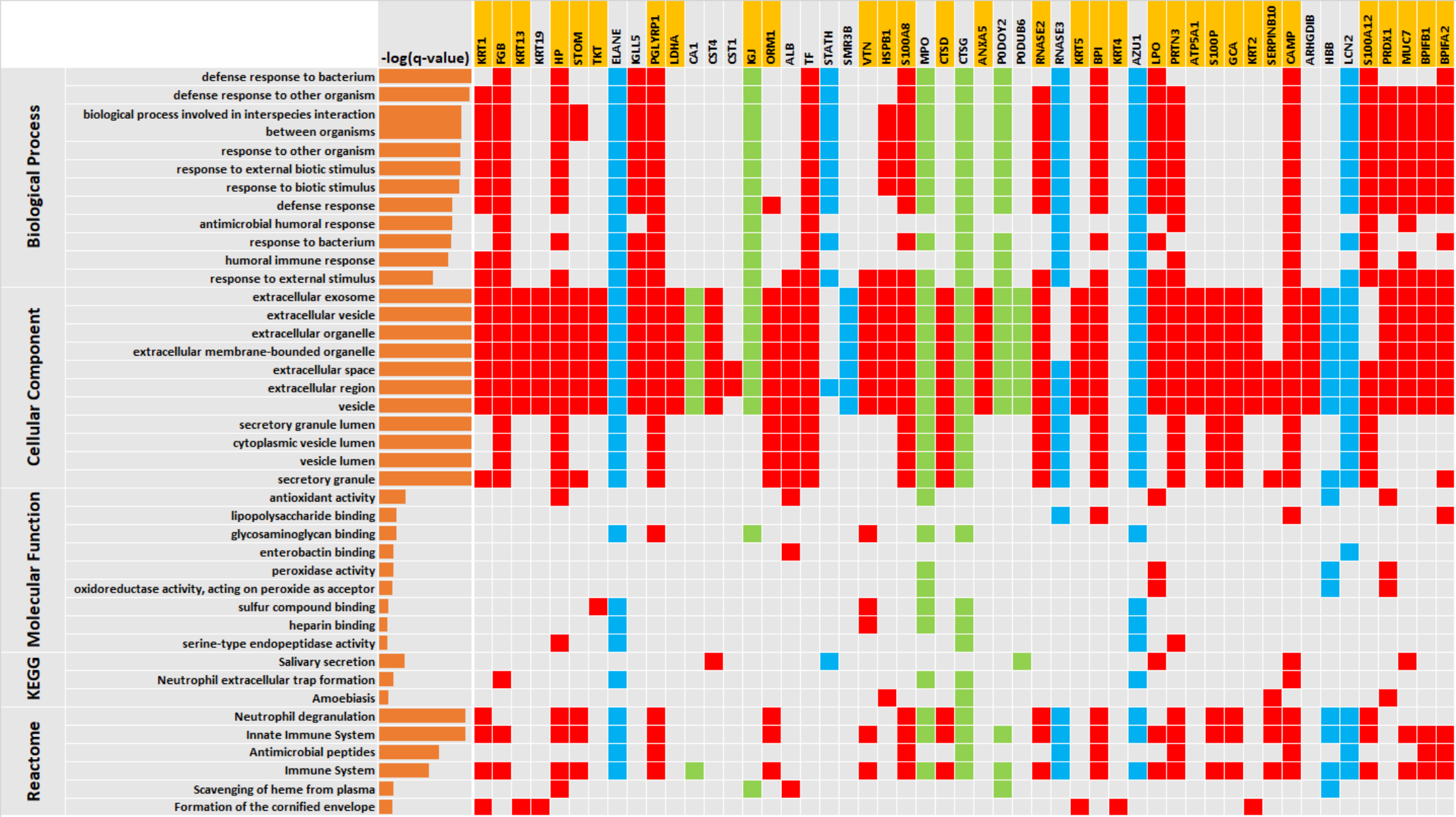


**Additional file 1: Figure S3.** Visualizing enrichment analysis of the most common proteins identified in the LIP group considers Biological Process GO, Cellular Component GO, Molecular Function GO, KEGG and Reactome terms with the highest significance (the highest values of -log(q-value)). The names of proteins marked with yellow colour point unique quantified proteins for the LIP group. Fill colours correspond with the type of regulation of protein: red - up-regulation of protein among the whole group, blue – down-regulation of protein, and green – level o regulation of protein is varied among the proteins in the group.


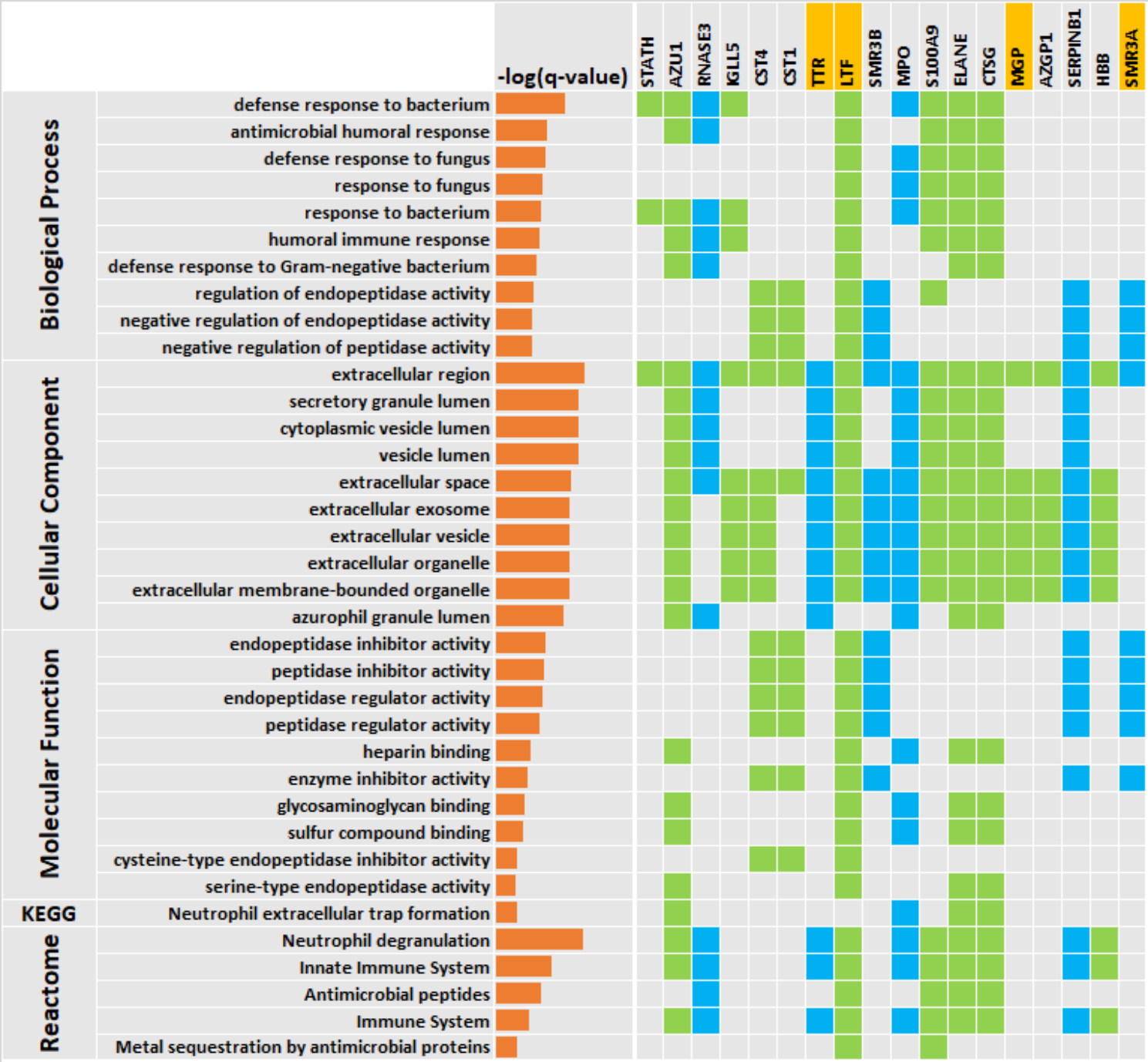


**Additional file 1: Figure S4.** Visualizing enrichment analysis of the most common proteins identified in the MIX group considers Biological Process GO, Cellular Component GO, Molecular Function GO, KEGG and Reactome terms with the highest significance (the highest values of -log(q-value)). The name of the protein marked with a yellow colour point is a unique quantified protein for the MIX group. Fill colours correspond with the type of regulation of protein: red - up-regulation of protein among the whole group, blue – down-regulation of protein, and green – level o regulation of protein is varied among the proteins in the group.


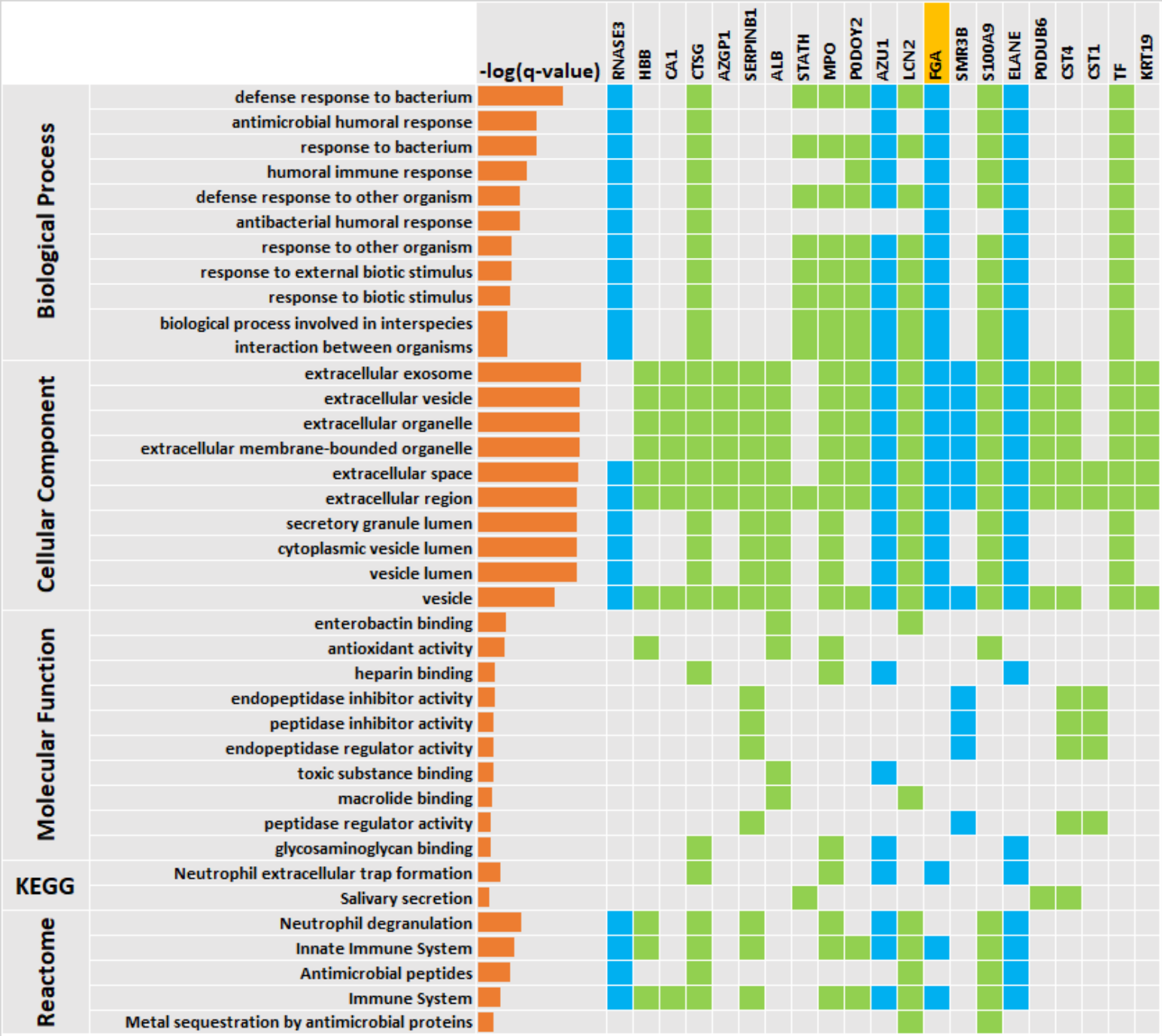


**Additional file 1: Figure S5.** PCA analysis of all the clinical sialolith and pooled samples were used in relative quantitative analysis. Data were normalised using the total area sums (TAS) approach, and technical replicates of samples were averaged.


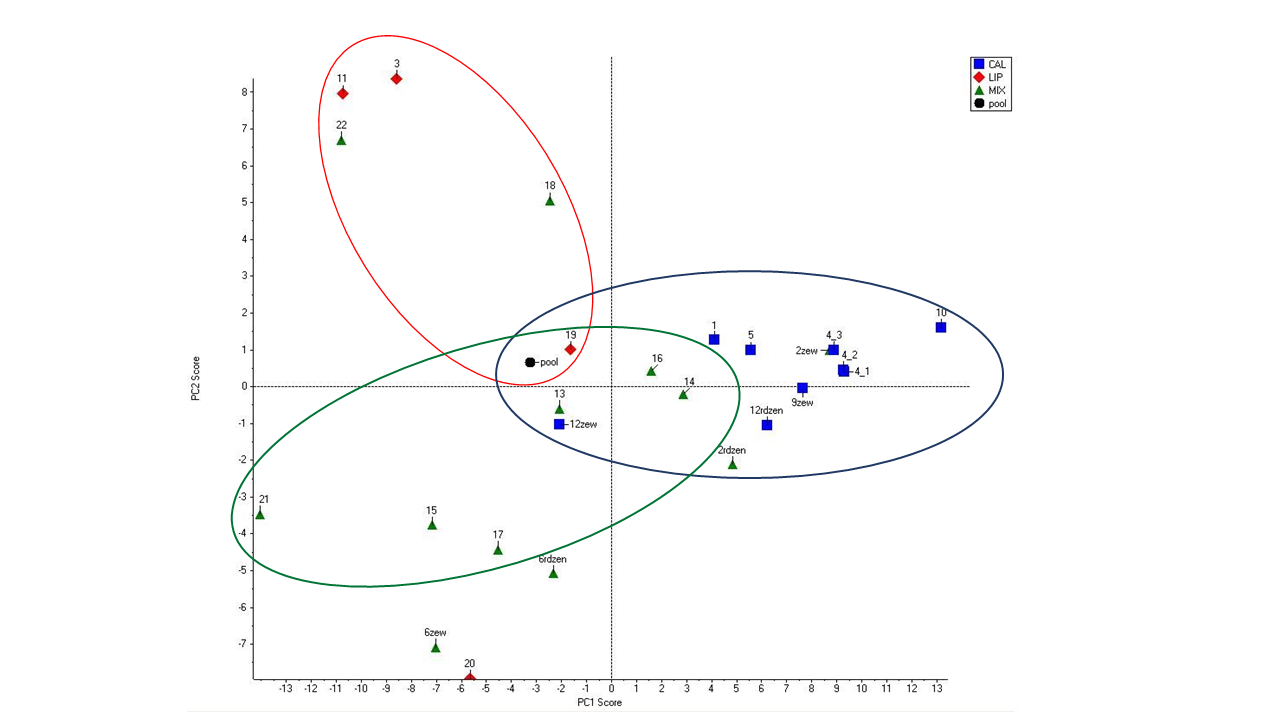


**Additional file 1: Table S5.** A table presents the level of regulation of proteins, which is statistically significant (q<0,05) and the log2FC≥0,45 for up-regulated proteins and log2FC≤-0,45 for down-regulated proteins for more than 50% of proteins from each group and are typical for paired sialolith groups. Red colour corresponds with the up-regulation of protein among the whole group, blue – is the down-regulation of protein, and green – level o regulation of protein regulation varies among the group proteins.

**Additional file 1: Figure S6.** Heatmap presents the level of regulation of proteins, which are statistically significant (q<0,05) and the log2FC≥0,45 for up-regulated proteins and log2FC≤-0,45 for down-regulated proteins for more than 50% of samples standard for CAL, LIP and MIX groups. The red colour corresponds with values of log(FC) for up-regulated proteins, and the blue corresponds with values of log(FC) for down-regulated proteins.


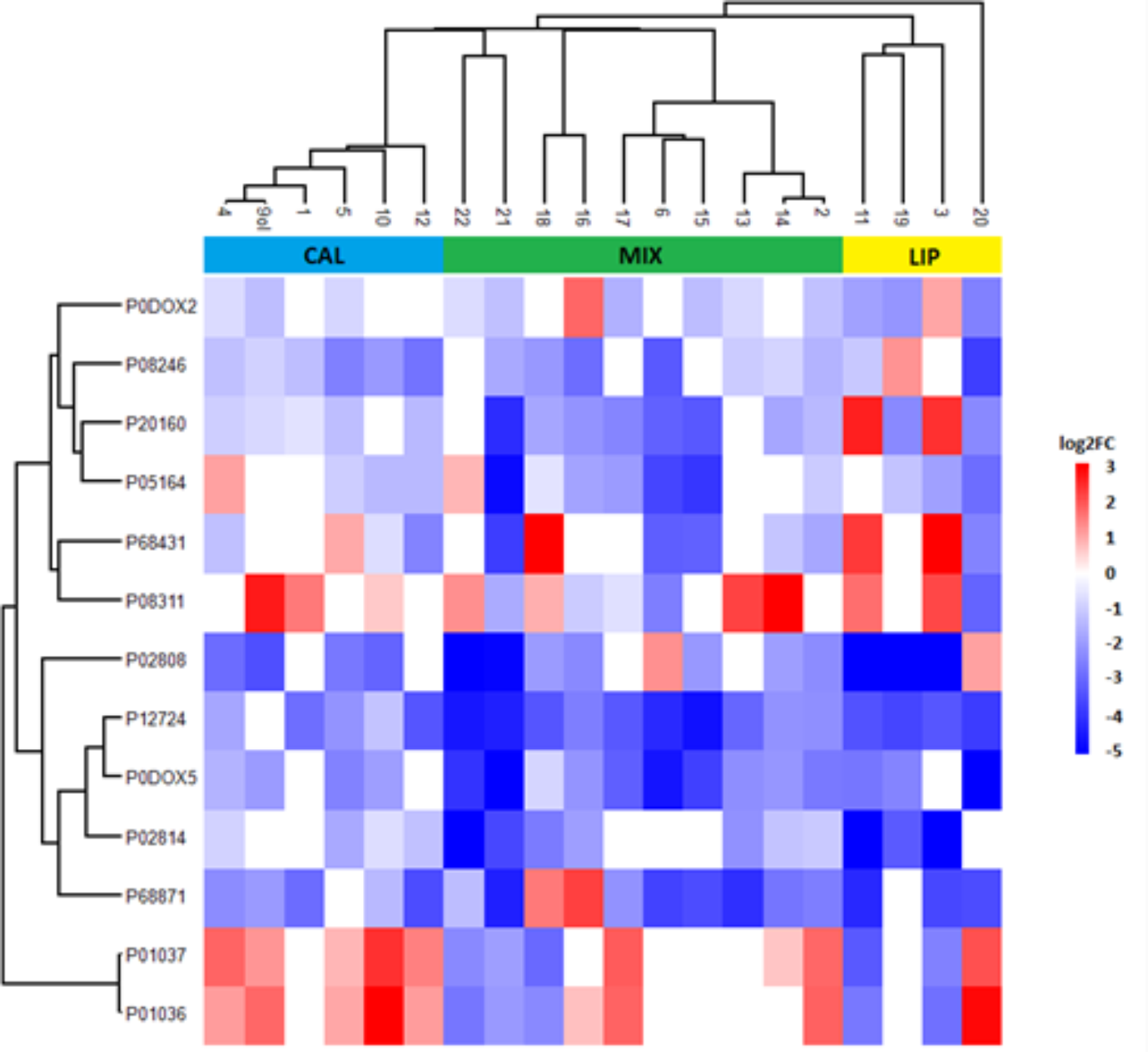


**Additional file 1: Figure S7.** The Cytoscape visualisation of the STRING-generated network is composed of experimentally verified protein-protein interactions among the proteins common for CAL, LIP and MIX groups. The size of nodes corresponds with the frequency of occurrence of protein among all of the samples in the group; bigger nodes represent higher frequency, and smaller – ones represent lower frequency. Fill colours correspond with the type of regulation of protein: red - up-regulation of protein among the whole group, blue – down-regulation of protein, and green – level of regulation of protein is varied among the proteins in the group.


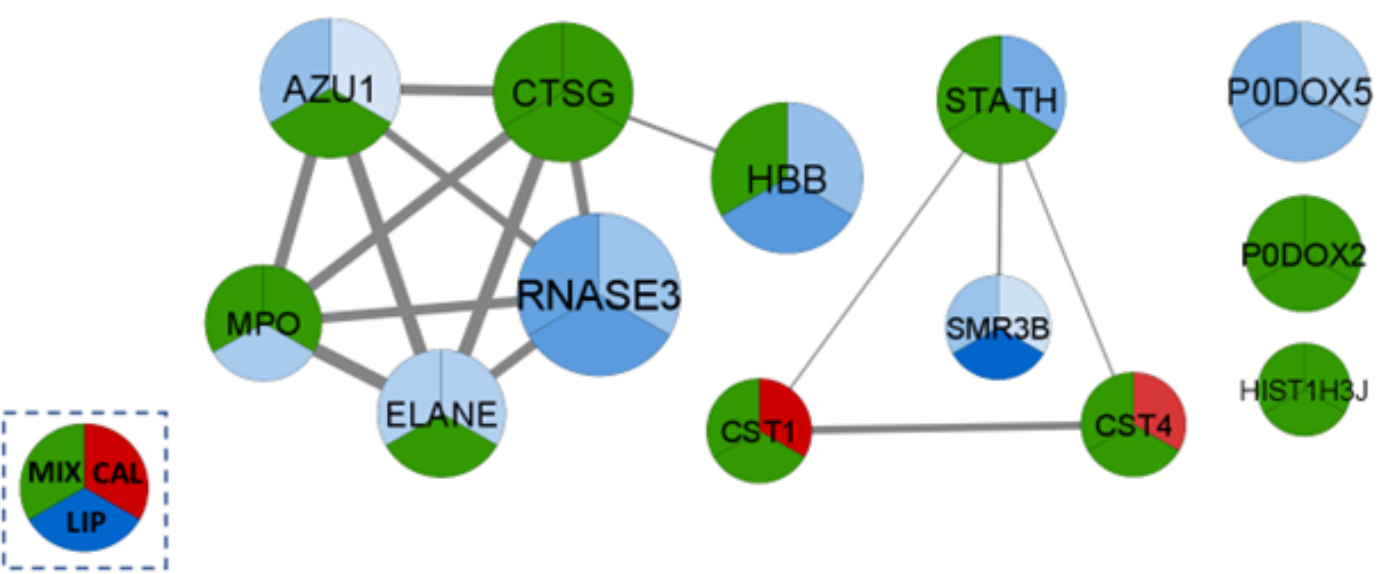


**Additional file 1: Figure S8.** The visualization of enrichment analysis of the proteins common for CAL, LIP and MIX groups. Fill colours correspond with the type of regulation of protein: red - up-regulation of protein among the whole group, blue – down-regulation of protein, and green – level o regulation of protein is varied among the proteins in the group.


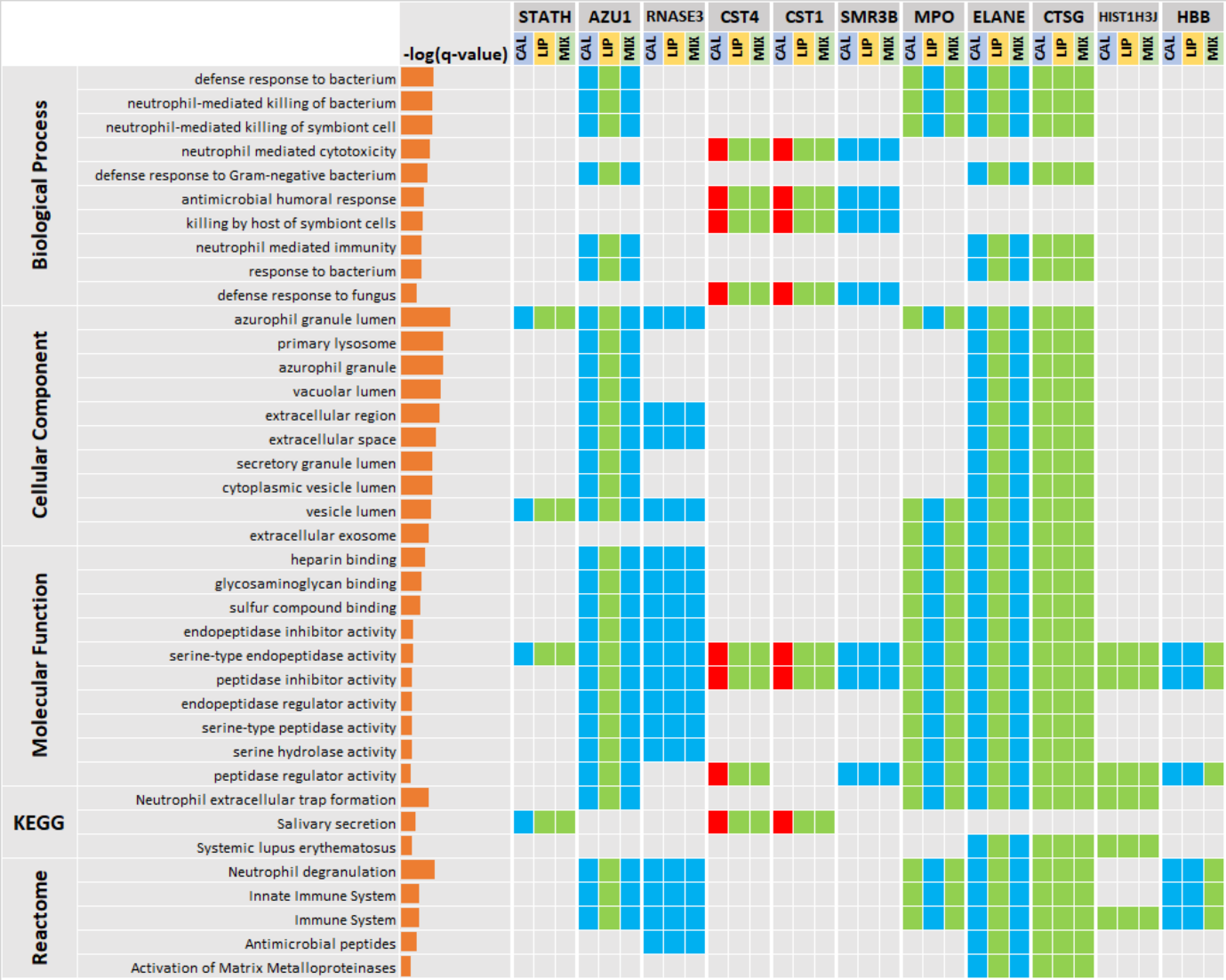

Supplement: Supplementary file 1 — Additional file 1: Figure S1. Chart presenting the numbers of human and bacterial proteins groups at 1% FDR with minimum 2 different peptides (bar graph) and the numbers of bacteria species from which the bacterial proteins were detected in each sample (blue curve). Table S1. All of the bacteria species, from which the proteins at 1% FDR with minimum 2 different peptides, were detected. Table S2. A table with the number of up-regulated and down-regulated proteins for each clinical sample from the CAL group, which are statistically significant (q<0.05) and the log2FC≥0.45 for up-regulated proteins and log2FC≤-0.45 for down-regulated proteins. Table S3. A table with the number of up-regulated and down-regulated proteins for each clinical sample from the LIP group, which are statistically significant (q<0.05) and the log2FC≥0.45 for up-regulated proteins and log2FC≤-0.45 for down-regulated proteins. Table S4. A table with the number of up-regulated and down-regulated proteins for each clinical sample from the MIX group, which are statistically significant (q<0.05) and the log2FC≥0.45 for up-regulated proteins and log2FC≤-0.45 for down-regulated proteins. Figure S2. Visualizing enrichment analysis of the most common proteins identified in the CAL group considers Biological Process GO, Cellular Component GO, Molecular Function GO, KEGG and Reactome terms with the highest significance (the highest values of -log(q-value)). The names of proteins marked with yellow colour point unique quantified proteins for the CAL group. Fill colours correspond with the type of regulation of protein: red—up-regulation of protein among the whole group, blue – down-regulation of protein, and green—level o regulation of protein is varied among the proteins in the group. Figure S3. Visualizing enrichment analysis of the most common proteins identified in the LIP group considers Biological Process GO, Cellular Component GO, Molecular Function GO, KEGG and Reactome terms with the highest signif [file 12014_2023_9402_MOESM1_ESM.docx]
